# Investigating the Effects of Brainstem Neuronal Adaptation on Cardiovascular Homeostasis

**DOI:** 10.3389/fnins.2020.00470

**Published:** 2020-05-20

**Authors:** James H. Park, Jonathan Gorky, Babatunde Ogunnaike, Rajanikanth Vadigepalli, James S. Schwaber

**Affiliations:** ^1^Department of Pathology, Anatomy and Cell Biology, Jefferson Medical College, Daniel Baugh Institute for Functional Genomics and Computational Biology, Thomas Jefferson University, Philadelphia, PA, United States; ^2^Department of Chemical and Biochemical Engineering, University of Delaware, Newark, DE, United States; ^3^Institute for Systems Biology, Seattle, WA, United States

**Keywords:** baroreceptor reflex, neuronal adaptation, vagus, accentuated antagonism, systolic heart failure

## Abstract

Central coordination of cardiovascular function is accomplished, in part, by the baroreceptor reflex, a multi-input multi-output physiological control system that regulates the activity of the parasympathetic and sympathetic nervous systems via interactions among multiple brainstem nuclei. Recent single-cell analyses within the brain revealed that individual neurons within and across brain nuclei exhibit distinct transcriptional states contributing to neuronal function. Such transcriptional heterogeneity complicates the task of understanding how neurons within and across brain nuclei organize and function to process multiple inputs and coordinate cardiovascular functions within the larger context of the baroreceptor reflex. However, prior analysis of brainstem neurons revealed that single-neuron transcriptional heterogeneity reflects an adaptive response to synaptic inputs and that neurons organize into distinct subtypes with respect to synaptic inputs received. Based on these results, we hypothesize that adaptation of neuronal subtypes support robust biological function through graded cellular responses. We test this hypothesis by examining the functional impact of neuronal adaptation on parasympathetic activity within the context of short-term baroreceptor reflex regulation. In this work, we extend existing quantitative closed-loop models of the baroreceptor reflex by incorporating into the model distinct input-driven neuronal subtypes and neuroanatomical groups that modulate parasympathetic activity. We then use this extended model to investigate, via simulation, the functional role of neuronal adaptation under conditions of health and systolic heart failure. Simulation results suggest that parasympathetic activity can be modulated appropriately by the coordination of distinct neuronal subtypes to maintain normal cardiovascular functions under systolic heart failure conditions. Moreover, differing degrees of adaptation of these neuronal subtypes contribute to cardiovascular behaviors corresponding to distinct clinical phenotypes of heart failure, such as exercise intolerance. Further, our results suggest that an imbalance between sympathetic and parasympathetic activity regulating ventricular contractility contributes to exercise intolerance in systolic heart failure patients, and restoring this balance can improve the short-term cardiovascular performance of these patients.

## Introduction

Cardiovascular homeostasis is maintained by complex multi-input, multi-output physiological control systems that interact with one another to regulate various cardiovascular parameters. One such system, the baroreceptor reflex, provides negative feedback control of blood pressure by modulating such physiological parameters as heart rate and cardiac output via interactions among brainstem nuclei that coordinate the parasympathetic and sympathetic branches of the autonomic nervous system. It is well established that coordination between the parasympathetic and sympathetic branches is central to maintaining cardiovascular homeostasis. By the same token, an imbalance between these two branches is a classic feature of cardiovascular disease such as heart failure with reduced ejection fraction (HFrEF) (Olshansky et al., 2008; Bibevski and Dunlap, 2011; Kishi, 2012). Decreased parasympathetic and increased sympathetic activity define the autonomic imbalance associated with many of the symptoms seen in HFrEF, including disease progression, exercise intolerance, ventricular remodeling, and arrhythmias. Understanding the putative interactions involved in this autonomic imbalance is needed to help elucidate the (patho)physiological and (dys)functional adaptations in HF.

Traditionally, parasympathetic activity, mediated by the vagus nerve, is known for its role in decreasing heart rate as well as playing an anti-inflammatory role in cardiovascular health (De Ferrari and Schwartz, 2011). However, anatomical and physiological evidence shows that parasympathetic activity affects multiple cardiac functions in addition to heart rate, including myocardial relaxation (lusitropy) and conductivity (dromotropy) (Coote, 2013). Similarly, multiple reports have demonstrated that parasympathetic activity plays a direct role in contractility (inotropy) as well (Lewis et al., 2001; Fukuda et al., 2015; Machhada et al., 2015; Dyavanapalli et al., 2016; Gourine et al., 2016). Consequently, parasympathetic activity plays multiple influential roles in cardio-protection beyond decreasing heart rate and mitigating sympathetic arousal (Kobayashi et al., 2013), so much so that vagal nerve stimulation has become a central focus in several clinical trials for HF therapy (Gold et al., 2016; De Ferrari et al., 2017; Konstam et al., 2019). Although the results of these clinical trials have been mixed (Hanna et al., 2018), understanding the role of vagal nerve stimulation in cardiac health continues to be an area of significant interest. This is evidenced by the NIH Common Fund SPARC program, which has the objective of discovering how to emulate the positive effects of vagal stimulation on the periphery, including the heart. Therefore, understanding the coordination between parasympathetic (vagal) and sympathetic control across *multiple* cardiac functions within the larger context of the baroreceptor reflex and cardiovascular system would provide deeper insight into how autonomic regulation affects cardiovascular performance in health and disease such as HFrEF (Olshansky et al., 2008; Bibevski and Dunlap, 2011; Kishi, 2012).

Despite extensive experimental investigation into and characterization of the baroreceptor reflex (i.e., baroreflex), understanding the behavior of this physiological control system as a whole remains a challenge. Some of the main barriers to understanding this system are attributable to the non-linear interactions among the individual components of this system. Moreover, single-cell analysis within the brain has shown that individual neurons are transcriptionally, and thus functionally, distinct from one another (Kim and Eberwine, 2010; Eberwine and Bartfai, 2011; Eberwine and Kim, 2015; Zeisel et al., 2015). Such transcriptional and functional heterogeneity, which is pervasive throughout the brain (Chiu et al., 2014; Park et al., 2014; Darmanis et al., 2015), complicates the task of understanding how functionally distinct neurons within and across brain nuclei organize and interact to regulate cardiovascular function.

To elucidate this complexity, we previously performed single-cell analysis of neurons belonging to the nucleus tractus solitarius (NTS), a brainstem nucleus involved in cardiovascular regulation (Park et al., 2014). Specifically, we profiled the transcriptional state of NTS neurons responding to a pharmacologically induced hypertensive challenge in a rat animal model. These neurons were selected based on their neuroanatomical location and FOS expression, a protein marker indicative of neuronal activity. Our results revealed an underlying molecular organizational framework in which the transcriptional states of an individual neuron align with the synaptic inputs received. Consequently, these neurons form distinct neuronal subtypes that populate a transcriptional landscape defined by graded, correlated gene expression. Further quantitative modeling and analysis of the transcriptional regulatory relationships underlying the transcriptional state of these brainstem neurons showed that distinct regulatory networks subtend these neuronal subtypes (Park et al., 2015). Taken together, these findings suggest that the transcriptional heterogeneity observed across brainstem neurons is due in part to the adaptation of individual neurons to inputs they receive over the course of the brainstem’s post-developmental history.

Motivated by these results, we postulate an alternative functional organization of NTS neurons involved in the baroreflex – neuronal subtypes, informed by the synaptic inputs received, respond and adapt to afferent signals relaying information on the state of cardiovascular system to maintain robust regulation of physiological functions. We therefore hypothesize that the presence of distinct neuronal subtypes and the adaptation of neuronal subtypes support robust (coordinated) biological function through graded cellular responses. To test this hypothesis, we use mathematical modeling to examine the functional impact of neuronal adaptation on cardiovascular function in health and disease. Here, we developed a mathematical model, based upon previous quantitative models of baroreflex regulation of cardiovascular function (Ursino et al., 1994; Rose and Schwaber, 1996; Ursino, 1998; Magosso and Ursino, 2002; Olufsen et al., 2006). We extend upon these models by adding multiple functions representing distinct neuroanatomical subtypes (Park et al., 2014) and brainstem nuclei involved in regulating parasympathetic (vagal) activity controlling cardiovascular function (Lewis et al., 2001; Coote, 2013; Fukuda et al., 2015; Machhada et al., 2015; Dyavanapalli et al., 2016; Gourine et al., 2016). The result is a detailed, extended model of the functional and neuroanatomical organization underlying parasympathetic (vagal) activity within the larger context of the baroreflex and cardiovascular homeostasis, which we used to investigate aspects of autonomic (dys)regulation and its contribution to cardiovascular health and the multifactorial clinical phenotypes associated with HFrEF.

To provide the reader with the most pertinent information and results regarding developing of the extended model and simulation analysis, the paper is organized into the following sections: (1) Prior models, which provides a brief background of previous work that formed the basis of the extended model, (2) Qualitative description of the extended model structure, (3) Extended model parameter estimation, which describes the process by which specific parameters values were selected, (4) Methods, which describes the various simulations performed and the sensitivity analysis methodology used, (5) Results, and (6) Discussion.

## Prior Models

The extended model primarily builds upon an earlier quantitative model developed by Ursino (1998) and its subsequent iteration (Magosso and Ursino, 2002). These models are of particular interest because they are comprehensive, not only in the sheer number of components of the cardiovascular system included in the model, but also in the extent to which each physiological component is modeled. For example, hemodynamic behavior throughout the cardiovascular systems is modeled using distinct capacitances, resistances, and unstressed volumes corresponding to different regions of the body. Multiple compartments of the heart were defined by distinct physiological parameters, allowing the model to capture the distinct contributions of the left and right aorta and ventricles in pulsatile flow affecting hemodynamics of the cardiovascular system. And of major importance for the development of the extended model was the inclusion of autonomic regulation of vascular resistance and cardiac function, i.e., heart rate, to reflect the full non-linear, antagonistic effect of sympathetic and parasympathetic activity on organ function. This antagonistic effect is observed most notably during the simultaneous regulation of heart rate by sympathetic and parasympathetic branches of the autonomic nervous system (ANS). Parasympathetic activity, which reduces heart rate, has a much larger inhibitory effect in the presence of sympathetic activity (Levy and Zieske, 1969), representing an *accentuated* antagonism affecting heart rate and ventricular elastance (Degeest et al., 1965; Levy and Zieske, 1969; Henning et al., 1990; Coote, 2013).

**FIGURE 1:**
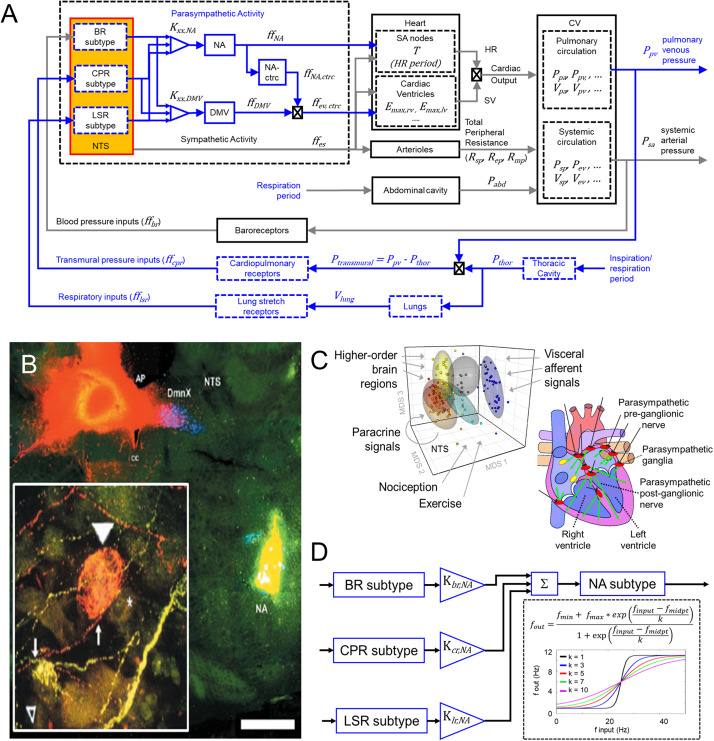
Modeling input-driven neuronal subtypes in central regulation of cardiovascular system. **(A)** The control block diagram of the cardiovascular system and its components incorporated into a closed-loop model, represents the flow of information involved in the autonomic regulation of the cardiovascular system. Components outlined in blue represent the new components incorporated into the model. Note that inspiration/respiration period is a parameter that changes depending on the modeled state (i.e., nominal or the exercise-challenged state). Several model parameters were included to indicate some of the parameters associated with the physiological components included in the model. Details of parameters are included in the Supplemental Text. BR, baroreceptor; CPR, cardiopulmonary receptor; LSR, lung stretch receptor; NA, nucleus ambiguus; NA-ctrc, nucleus ambiguus affecting ventricular elastance (a surrogate for contractility); DMV, dorsal motor nucleus of the vagus; *K*_*xx,YYY*_, gain constants for corresponding xx-subtype input affecting YYY-subgroup; *ff*, firing frequency; *E*_*max,rv/lv*_, maximum elastance of right ventricle (rv)/left ventricle (lv); *R*_*sp/ep/mp*_, resistance of splanchnic (sp), extrasplanchnic (ep), and lower body compartments (mp); *P*_*abd*_, abdominal pressure; *P*_*pa/pv*_, pressure of pulmonary arteries (pa) and pulmonary veins (pv); *P*_*sp/ev*_, pressure of splanchnic veins and extrasplanchnic veins; *V*_*sp/ev*_, volume of splanchnic and extrasplanchnic compartments; *P*_*thor*_, pressure of thoracic compartment. **(B)** Fluorescent imaging of brain stem and basket cell in the heart showing direct connection between Nucleus Ambiguus (NA) and cardiomyoctye. Image modified from Cheng et al. (2004). This and similar evidence support the functional connectivity included in the control block diagram shown in **(A)**. **(C)** *Left* – multidimensional scaling plot visualizing similarities among the transcriptional (i.e., functional) state of neurons. Neuronal functional state is driven by received inputs, thus neurons receiving similar input-types share similar functional states. Image modified from Park et al. (2014). *Right* – schematic indicating the multiple regions of the heart innervated by the vagus. Image modified from Coote (2013). **(D)** Specific input-driven neuronal subtypes/neuron-types generate outputs, which subsequently are weighted based on their respective gain (*K_*xx,NA*_*, refer to Supplementary Table S23 for values). These weighted values are fed downstream to the appropriate process control block. The control block representing the NA neurons receives inputs from multiple neuron subtypes (including neurons driven by baroreceptor inputs, *BR subtype*), cardiopulmonary receptor inputs (*CPR subtype*), and lung stretch receptor inputs (LSR subtype). The firing frequency outputs generated by these subtypes are added together to form the overall firing frequency input to the NA neurons. Sigmoidal functions represent the input-output transfer functions for both the neuronal subtypes and neuronal populations added in the brainstem. “*” is meant to represent the multiplication operator.

We build upon these models by including additional model components (Figure 1A) to represent more precisely the neuronal components involved in parasympathetic regulation of cardiovascular functions underlying healthy and diseased states, specifically HFrEF and exercise intolerance, an associated clinical phenotype of this disease. Multiple confounding factors underlie this complex disease, e.g., persistent ischemic stress, which pose challenges in quantitatively modeling HFrEF. However, given that multiple physiological cardiovascular components were previously included in the original model (Ursino, 1998) and the autonomic-related modifications capture heart-brain axis in greater detail, the extended model is well suited to investigate the role of autonomic dysregulation in cardiovascular dysfunction in HFrEF and exercise intolerance. By simulating neuronal adaptation, which results from the multiple inputs and changes brought on by HF, we investigate the consequence(s) of these mal-adaptations and their putative contributions to the observed cardiovascular behaviors defining HFrEF and exercise intolerance.

## Qualitative Description of Extended Model Structure

As the control center regulating autonomic activity, the NTS and its neuronal subtypes interact with one another and the NA and DMV to regulate parasympathetic activity. Ample experimental evidence indicates that the NA and DMV affect distinct cardiac functions and are the sources of vagal efferent outflow (Wang et al., 2001; Bibevski and Dunlap, 2011; Chapleau and Sabharwal, 2011; Mastitskaya et al., 2012; Coote, 2013; Machhada et al., 2015). Consequently, they represent two key targets of continuing investigation of parasympathetic regulation of cardiac function. Thus, to extend the prior models developed by Ursino (1998), and Magosso et al. (2002) to reflect more accurately the physiological components regulating parasympathetic activity, we included additional model components that represent: (1) NTS neuronal subtypes defined by synaptic input types (Figures 1B,C, Park et al., 2014, 2015), (2) the *nucleus ambiguus* (NA), and (3) the *dorsal motor nucleus of the vagus* (DMV).

### Functional Connectivity in the Brainstem

**FIGURE 2:**
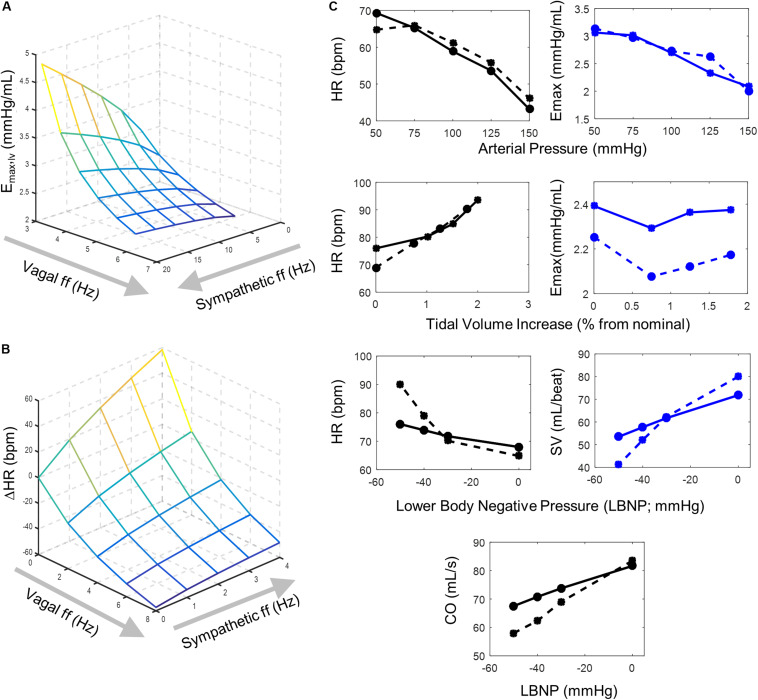
Model behavior and parameter fitting. Simulation outputs (solid lines) generated using the final parameter sets determined from parameter selection process and corresponding experimental data (dashed lines) used to evaluate model fit. **(A)** A surface plot representing simulated changes in left ventricular elastance resulting from simultaneous vagal and sympathetic activity. Each intersection on the surface plot represents a simulated vagal and sympathetic firing frequency condition. The accentuated antagonistic effect of vagal and sympathetic activity is indicated by the increasing inhibitory effect vagal activity has at higher sympathetic activity levels **(B)** Surface plot representing simulated changes in heart rate at different combinations of simultaneous vagal and sympathetic ff. Each intersection on the surface plot represents a simulated condition. Simulations that used the final parameters selected to simulate values in **(A)** were run under open-loop conditions to mimic the open-loop experimental conditions from which the experimental data was collected (Levy and Zieske, 1969), which originally demonstrated the accentuated antagonistic relationship that vagal and sympathetic ff have on heart rate. **(C)** *Top row –* simulated heart rate and ventricular elastance (solid lines) generated under open-loop conditions (Supplementary Figure S1A) using parameters selected for sigmoidal functions representing the (1) baroreceptor input-driven subtype, (2) the NA, (3) NA_contractility_, and (4) the DMV neuron-type and corresponding experimental data (dashed lines) collected by Suga et al. (1976). Heart rate and ventricular elastance values of 61.2 bpm and 2.695 mmHg/mL, respectively, were used as baseline values during the parameter selection process (Ursino, 1998). *Middle row –* simulated heart rate and ventricular elastance generated under open-loop conditions using parameters selected for the lung stretch receptor neuronal subtype and corresponding data measuring the effects of lung volume on heart rate and elastance (Hainsworth, 1974; Greenwood et al., 1980). For parameter estimation purposes, baseline heart rate and elastance values of 84.6 bpm and 2.392 mmHg/mL, respectively, were used (Ursino, 1998). Moreover, the parameter selection process for the sigmoid function representing the input-output behavior of the lung stretch receptor subtype relied on simulations using parameters selected for the NA, NA_contractility_, DMV, and baroreceptor neuronal subtypes (top row). *Bottom row* – Simulated heart rate, stroke volume, and cardiac output, generated under closed-loop model conditions, and corresponding experimental data measured in subjects sitting in a lower body negative pressure (LBNP) chamber (Frey, 1986). Parameter selection for the cardiopulmonary neuronal subtype was based on simulations using parameters identified for the NA, NA_contractility_, DMV, baroreceptor (top row), and lung stretch receptor-neuronal subtypes (middle row).

Physiological experiments have shown that the NA and DMV modulate specific cardiac effector functions: the NA modulates heart rate and, to a lesser extent, ventricular contractility while the DMV primarily affects contractility, although to a lower extent than sympathetic activity (Dampney, 1994; Cheng et al., 1999; Wang et al., 2001; Mastitskaya et al., 2012; Machhada et al., 2015). NA and DMV connectivity included in this extended model are based on experimentally established electrophysiological characteristics of these nuclei. For instance, firing patterns of the NA have been shown to align with the respiratory cycle, suggesting that neuronal populations within the NA are in part dependent upon synaptic signals relaying information related to respiration. Firing patterns within the DMV have been shown to be less dependent on respiration (Jones et al., 1998; Coote, 2013), suggesting respiratory rhythms have a diminished influence on the DMV. Based on this information, we propose a connectivity structure relating brainstem neuronal components to physiological functions within our model that is consistent with these experimental findings (Figure 1A). One caveat to the connectivity shown in Figure 1A: although activity of DMV neurons is less dependent on respiration, a connection between the lung stretch receptors and DMV is included in order to mimic the relationship between lung tidal volume and ventricular elastance, which is mediated by lung stretch receptors (Figure 2C, middle row, *E*_*max*_ vs. tidal lung volume), given that the DMV is the main regulator of ventricular elastance, a surrogate measure for ventricular contractility, in the context of this model.

### NTS, NA, and the DMV Function and Role in Parasympathetic (Vagal) Outflow

Because we are primarily interested in understanding what effects distinct neuronal subtypes (and adaptation of these subtypes) may have on parasympathetic outflow, we incorporated additional components into the model developed by Ursino (1998) and Magosso et al. (2002). These models provide a framework within which to investigate how a synaptic input-driven molecular organization (Park et al., 2014) affects cardiovascular function by evaluating via simulation, steady-state hemodynamic and cardiovascular behaviors under healthy (nominal), diseased, and acutely challenged conditions. We used transfer functions to model the dynamic response of three neuronal subtypes that generate afferent signals relaying specific information about blood pressure and pulmonary state affecting cardiovascular function: (1) carotid baroreceptors, (2) cardiopulmonary receptors, and (3) SAR lung-stretch receptors. These three subtypes were chosen based on several factors, including availability of physiological data and their inclusion within previous models (Ursino, 1998; Magosso et al., 2002). Similarly, transfer functions were included to represent the signal processing and vagal regulatory functions of the NA and DMV. Because the NA affects both heart rate and contractility (Dampney, 1994; Cheng et al., 1999; Wang et al., 2001; Mastitskaya et al., 2012; Machhada et al., 2015), two separate control blocks (NA and NA_contractility_) are included to represent these distinct influences of the NA (Figure 1). The input-output relationship in each transfer function block is represented as a sigmoidal function, to capture appropriately the saturating firing behavior of neurons (in response to increasing input firing frequency) – a well-known characteristic of the baroreflex (Rogers et al., 1993; Ursino, 1998; Kawada et al., 1999). Because vagal outflow has been shown to increase monotonically as a function of activity in the carotid sinus nerve, with an upper saturation (Ursino, 1998), the following sigmoidal functions are used to describe the input-output relationship of these components:

(1) $$f_{o\,u\,t,j}=\frac{f_{\min,j}+f_{\max,j*\exp}\,\left(\frac{f_{i\,n\,p\,u\,t,j}-f_{m\,i\,d\,p\,t,j}}{k}\right)}{1+\exp\left(\frac{f_{i\,n\,p\,u\,t,j}-f_{m\,i\,d\,p\,t,j}}{k,j}\right)}$$

Here, *f*_*min,j*_ and *f*_*max,j*_ represent the firing frequency output range of the *j*th neuronal subtype or neuronal population. The variable *f*_*input,j*_ refers to the firing frequency input from the respective receptor-type that the specific neuronal subtype is responding to (being shaped by). The input firing frequency that leads to a half-maximal output is represented by *f*_*midpt,j*_ and reflects the range of inputs to which the specific subtype responds. Finally, *k*_*j*_ represents the “sensitivity” of the neuronal subtype to inputs received where larger *k*-values indicate lower input sensitivity, so that the response over a broad range of inputs is a gradual and relatively small increase in output, and smaller *k*-values indicate higher input sensitivity (sharper, more pronounced increase in output over the same range of inputs). A *k*-value of unity represents near-binary, “on-off” response profile (Figure 1D). This sigmoidal model was chosen because it requires fewer parameters and the parameters have physiological relevance and meaning.

### Efferent Outflow of the Sympathetic and Parasympathetic Nervous Systems

The extended model uses the same functions developed by Ursino (1998) to represent the relationship between afferent inputs relaying peripheral sensory information (e.g., arterial pressure) and sympathetic/parasympathetic efferent outflow. Briefly, an increase in the firing frequency of an afferent input (e.g., firing frequency generated by baroreceptors) results in a reduction in the firing frequency of sympathetic fibers, and a concomitant increase in the vagus nerve. The firing frequency of sympathetic fibers, which represents sympathetic efferent outflow, is modeled mathematically as a monotonically decreasing exponential curve as a function of afferent input firing frequency. Conversely, vagal firing frequency, which represents parasympathetic efferent outflow, is modeled as a monotonically increasing exponential curve, with an upper saturating limit (Ursino, 1998). Additionally, distinct time delays known to occur due to signal processing in the sympathetic and parasympathetic branches are represented as part of the time-delayed response of effector functions to sympathetic and parasympathetic efferent outflow, respectively (Ursino, 1998; Magosso and Ursino, 2002).

### Autonomic Regulation of Effector Functions – Heart Rate and Ventricular Elastance

Sympathetic and parasympathetic efferent activities regulate heart rate and elastance simultaneously, having an accentuated antagonistic effect on these cardiac functions. This means that parasympathetic activity has a much larger inhibitory effect in the presence of sympathetic activity. To model the effect of this relationship quantitatively, the extended model includes an explicit and direct relationship between sympathetic and parasympathetic activity and heart period (the inverse of heart rate) rather than heart rate itself (Ursino, 1998). Heart period is calculated as the linear sum of the positive changes induced by parasympathetic activity and the negative changes induced by sympathetic activity to heart period (Ursino, 1998). The response of heart period to sympathetic activity is characterized as a monotonic function with linear first-order dynamics and a time constant defining the characteristic response time of heart period. Conversely, heart period has been shown to increase linearly with parasympathetic efferent activity (Parker et al., 1984). Modeling heart period as opposed to heart rate provides an adequate approximation of heart rate over a range of sympathetic and parasympathetic activity levels (Figure 2C), which has been previously demonstrated experimentally (Levy and Zieske, 1969; Ursino, 1998).

Because maximum ventricular elastance (*E*_*max*_), the maximum slope of the pressure-volume relationship at the end of systole and a robust correlate to contractility (inotropy; Suga et al., 1976), is also regulated by sympathetic and parasympathetic activity in a manner similar to how heart rate is regulated, we modeled the autonomic regulation of elastance using a similar approach to that used for heart rate, i.e., using the inverse of elastance. Like heart period, the *inverse* of elastance (inverse elastance) is defined by the sum of the positive and negative changes caused by parasympathetic and sympathetic activity, respectively. Here, the response of *inverse elastance* to sympathetic activity follows a monotonic function with linear first-order dynamics and an elastance-specific time constant (Supplementary Text). Concomitantly, *inverse elastance* increases linearly with parasympathetic activity. Thus, *inverse elastance* is determined by the sum of the responses to parasympathetic and sympathetic activities. By modeling the inverse of elastance in this manner, as in heart rate, we were able to simulate the accentuated antagonistic effect that simultaneous sympathetic and parasympathetic regulation has on ventricular contractility, qualitatively demonstrated in Figure 2B. Further details regarding these functions are provided in Supplementary Text.

### Hemodynamic and Cardiovascular Response to Exercise

To investigate cardiovascular performance of healthy and systolic heart failure patients, we created a separate “exercise-response” version of the extended model, which includes several structural changes that reflect the physiological responses occurring during exercise. In a clinical setting, clinicians assess how well a patient can tolerate exercise (i.e., exercise tolerance) by measuring cardiovascular performance during a pedaling cycle test. The sudden onset of exercise triggers multiple physiological responses, which includes, for example, a redistribution of blood throughout the body. Blood flow to the skeletal muscle in the legs increases, comprising ∼13% of total cardiac output (Magosso et al., 2002). Strong intramuscular contractions in the leg during exercise cause veins to collapse periodically (creating a “muscle pump” that modulates blood flow). Muscle vasodilation causes a decrease in systemic peripheral resistance, which counters the increase in systemic arterial pressure and consequently leads to a modest rise in arterial pressure. Furthermore, a commensurate increase in sympathetic activity and decrease in parasympathetic activity regulating the splanchnic (i.e., abdominal region) and cardiac compartments occur to meet the increased metabolic demand caused by the acute exercise challenge (Christensen and Galbo, 1983; Fisher, 2014). These changes in autonomic activity lead to an increase in heart rate and a large increase in cardiac output. Thus, to capture these physiological responses appropriately in the “exercise-response” version of the extended model, we made the following changes to the extended model: (1) added separate vascular compartments for resting and active muscles, (2) included a lower body muscle pump in which intramuscular pressure is characterized as a sinusoidal wave to mimic the periodic activity of the lower body during exercise, (3) decreased the inspiration/expiration ratio, which consequently changes thoracic pressure, and (4) included a positive offset to sympathetic activity during exercise. While each of these changes have been included in prior models (Ursino, 1998; Magosso et al., 2002; Magosso and Ursino, 2002), we have integrated these modifications into a single “exercise-response” model. Specific details are included in Supplementary Text.

### Simulation Platform

We developed the extended model on the SIMULINK (MathWorks) simulation platform because it provides a natural environment for modeling control systems whose components are represented as blocks interconnected by clearly identified inputs and outputs to each block. SIMULINK’s block-oriented framework therefore facilitates the modeling and analysis of *any* system – no matter how complex – that can be represented as an interconnected network of “blocks.” The ordinary differential equations in the blocks are solved using ODE15s and an error tolerance of 1e-3.

## Extended Model Parameter Estimation

Prior to investigating how an input-driven neuronal organization affects central regulation of cardiovascular function, first we had to determine values for model parameters that would produce simulation results matching established physiological behavior. As a starting point, for the portions of the extended model that were based upon prior models developed by Ursino (1998), and Magosso et al. (2002), we used the same parameter values as those used in the original model (Supplementary Text and Supplementary Tables S2–S23). The remaining parameter values were selected to produce simulations that best matched experimental data associated with nominal, diseased, and exercise-challenged states. Because we relied on multiple sources for experimental data to estimate model parameters, we summarized key experimental details of these experiments, e.g., model type used, number of replicates, and measured outputs, for reference (Supplementary Table S24).

### Brainstem-Associated Parameter Estimation (Nominal Conditions)

Given that the available experimental data are measurements of overall cardiac function in response to some experimentally induced sensory stimulus (e.g., increased arterial pressure), the use of multiple transfer functions to represent the dynamic behavior of the brainstem nuclei of interest raises the possibility of over-parameterization of the autonomic mechanisms regulating cardiovascular function. Hence, several distinct sets of parameter values can produce identical cardiovascular system behaviors and corresponding metrics quantifying that behavior like mean cardiac output (CO), stroke volume (SV), or heart rate (HR). Indeed, our previous findings indicate that brainstem neurons adapt to inputs in order to generate an appropriate physiological response to maintain a blood pressure set point (Park et al., 2014). Because brainstem neurons can exist in multiple functional states, it is unlikely that one unique set of parameters will be able to characterize the various functional states of these neurons sufficiently. Consequently, our aim is to identify sets of parameter values that describe plausible functional states of brainstem neurons and analyze how changes in these states, represented by changes in these parameter sets, affect cardiovascular homeostasis. Toward this end, we must identify biologically plausible parameter sets that describe the functional states of the neuronal subtypes and subpopulations of interest.

Thus, to address partially the issue of non-uniqueness of parameter estimates in this work, we constrained the possible parameter space to ensure that estimates were biologically feasible, i.e., comparable to reported firing frequencies of brainstem neurons or closely related sensory neurons. Thus, the constrained parameter space explored for each parameter was based on parameter values originally used by Ursino (1998) to describe the dynamic behavior of baroreceptors, whereby the possible parameter values were limited to within a ± 5-fold range of the corresponding baroreceptor parameter value. To explore the constrained parameter space, we selected 5000 random sets of parameter value estimates for each transfer function via Sobol sampling (Bratley and Fox, 1988; Hong and Hickernell, 2003; Joe and Kuo, 2003), which were used to simulate nominal, steady-state cardiovascular behavior. We chose 5000 sampling points for each parameter to explore thoroughly the range over which each parameter could exist. Parameter value estimates for the transfer functions of the neuronal subtypes and subpopulations in the brainstem were selected based on the sum of mean square errors (MSE) of HR, CO, SV, and/or ventricular elastance, depending on data availability.

We first selected relevant parameter value estimates that could be fit using data generated from experiments involving direct perturbations, which minimized the number of confounding afferent signals potentially affecting the physiological measure of interest. An example of a direct perturbation experiment would be the direct electrical stimulation of arterial baroreceptors to modulate heart rate and elastance (Suga et al., 1976). These direct perturbations corresponded to “open-loop” experimental conditions (i.e., in the absence of feedback). We then selected remaining parameter estimates using data generated from indirect perturbations, which were conducted under “closed-loop” experimental conditions where feedback effects modulating the response of the physiological measure of interest were not removed nor controlled for experimentally.

Following this approach, we first selected parameters for the transfer functions describing the dynamic firing responses of the baroreceptor input-driven subtype, NA, NA_contractility_, and DMV neuronal subtypes (Supplementary Figure S1A). We selected these parameter values that produced simulations in which the predicted mean heart rate (HR) and elastance response to changes in arterial pressure matched data generated by Suga et al. (1976) (Figure 2A top row). Next, we selected parameter values for the lung stretch receptor input-driven subtype that produced simulations in which the predicted HR and ventricular elastance response to changes in tidal lung volume (Supplementary Figure S1B) matched data from Greenwood et al. (1980) and Hainsworth (1974) (Figure 2A second row). For the final set of parameters for the transfer function describing the dynamic response of the cardiopulmonary receptors, we selected parameters values based on simulations that included the previously determined parameter values for the NA, NA_contractility_, DMV, baroreceptor- and lung stretch receptor-subtypes. In this instance, data were collected from adults placed in a lower body negative chamber, which causes blood to pool in the lower body and blood volume to decrease in the upper body. This, in turn, deactivates, or unloads, the cardiopulmonary receptors (Figure 2A bottom rows). Because the perturbation causing the changes in blood pressure were non-invasive and feedback effects affecting the cardiovascular system were not controlled for experimentally, a closed-loop model structure was used to fit parameter values (Supplementary Figure S1C). Parameter values were selected such that the predicted mean HR, SV, and CO resulting from changes in lower body negative pressure (Supplementary Figure S1C) matched experimental data for HR, SV, and CO. Details on how mean HR, SV, CO, and other quantifying metrics of hemodynamic and cardiovascular behavior were calculated from simulation outputs are detailed in the subsequent Methods section. The resulting sets of parameter values are available in Supplementary Tables S2–S23.

### Systolic Heart Failure Post Myocardial Infarction Parameter Estimation

**FIGURE 3:**
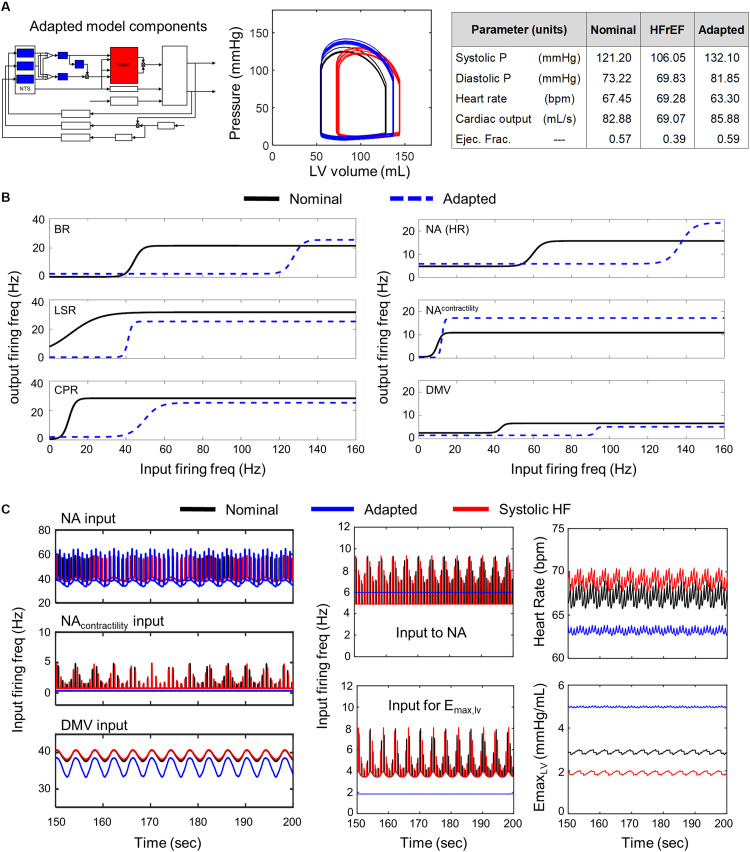
Effects of adaptation across brainstem neuronal subtypes in HFrEF condition. **(A)** *Left panel* – Schematic highlighting adjusted model components. *Middle panel* – simulated pressure-volume curve for baseline (black), heart failure (red), and adapted conditions (blue). *Right panel* – table summarizing some key cardiovascular outputs simulated by model. **(B)** *Left panel* – sigmoidal curves represent input-output functions characterizing BR-, LSR-, and CPR-neuronal subtypes. Baseline (black solid line) and adapted (blue dashed line) input-output functions are plotted. *Right panel* – input-output functions characterizing NA-, NA_contractility_, and DMV-neuronal groups. Baseline (black) and adapted (blue dash) input-output functions are plotted. **(C)** *Left panel* – Plots of input signal characteristics in brainstem due to adaptations occurring within the brainstem ff outputs are shown over the last 50 s of a 200 s simulation for the nominal (black), diseased (red), and adapted (blue) states. *Left top subpanel*: input ff received by the NA neuronal population that primarily affects heart rate. *Left middle panel*: input ff signal received by the NA neuronal population that primarily affects contractility. *Left bottom panel*: input ff signal received by the DMV neuronal population that affects contractility. *Middle top panel*: plot showing simulating vagal activity (*f*_*ev*_) modulating heart rate. The ff visualized represents the sum of the output signals generated by the NA_contractility_ and DMV neuronal populations. *Middle bottom*: vagal activity that modulates ventricular elastance. *Right top panel*: resulting heart rate. *Right bottom panel*: resulting elastance of the left ventricle.

Systolic heart failure (HFrEF) involves ventricular remodeling that leads to an enlarged left ventricle and reduced ventricular contractility, which results in an impaired ability to pump blood and a subsequent reduction in ejection fraction (EF), the fraction of blood pumped out of the left ventricle. Typically, patients suffering from systolic heart failure exhibit an EF below 0.5, the lower limit of what is considered a healthy EF (Paulus et al., 2007; Klein and De Ferrari, 2010; Schwartzenberg et al., 2012). Moreover, sympathetic activity increases in systolic heart failure patients as well. These physiological and autonomic changes cause the cardiovascular system to deviate from nominal, healthy behavior, which is characterized by an increase in end systolic volume/end diastolic volume, a decrease in CO, an overall decrease in cardiovascular health, and an increase in the risk of myocardial infarction recurrence. However, despite the multiple physiological changes that occur due to systolic heart failure, systolic and diastolic blood pressures remain within nominal ranges (Hung et al., 2010). Based on the physiological and autonomic changes that define systolic heart failure, we selected new values for parameters that define the physical characteristics and behavior of the heart. Using the same methodology devised to select parameter values for the brainstem-associated transfer functions, we identified a new set of parameter values that produced simulated cardiovascular behaviors matching those that characterize HFrEF patients (Figure 3A). The resulting parameter values used for subsequent simulation analyses are provided in Supplementary Table S23.

### Exercise Response (Exercise Intolerance) Parameter Estimation

Because the “exercise-response” version of the extended model incorporates modifications included across previous models (Ursino, 1998; Magosso et al., 2002; Magosso and Ursino, 2002), we used the same relevant parameters in the exercise-response model as those used in these previous models, where possible (Supplementary Text and Supplementary Table S15). For those parameters requiring new estimates, e.g., parameters for transfer functions modeling the dynamic responses of neurons in the brainstem, we used an approach similar to the one previously described [*Brainstem-associated parameter estimation (nominal conditions)*]. Here, we explored a parameter space that spanned a ± 5-fold range from nominal values of the brainstem-associated parameters identified previously to reproduce healthy behavior (Supplementary Tables S16–S22). Final parameter values (Supplementary Table S23) were selected such that simulated hemodynamic and cardiovascular behaviors matched those measured in healthy patients when their oxygen consumption levels (V_O__2_) reached 1L/min during the pedaling exercise test (Pawelczyk et al., 1992; Magosso et al., 2002).

When estimating parameters for exercise-intolerant systolic heart failure patients, we also selected different values for the parameters *G*_*s,Emax*_ and *G_*v,Emax*_*, in addition to those of the transfer functions modeling brainstem neuronal behavior. *G*_*s,Emax*_ and *G*_*v,Emax*,_ represent constant gain factors that quantify the effect that changes in sympathetic and parasympathetic activity have on ventricular elastance, respectively. These two parameters were included when selecting new parameter values in order to capture the deleterious effect that systolic heart failure has on the autonomic regulation of the heart. Parameter values (Supplementary Table S23) were selected such that simulated hemodynamic and cardiovascular behaviors matched the physiological responses measured experimentally in systolic heart failure patients with HFrEF when their V_O__2_ levels were approximately 1L/min during the pedaling exercise test (Dhakal et al., 2015).

## Methods – Simulation Approaches and Sensitivty Analysis

### Simulating and Quantifying Healthy and Heart Failure Physiological Conditions

Following parameter selection, we first performed simulations to demonstrate the extended model’s ability to reproduce hemodynamic and cardiovascular behaviors consistent with healthy adult physiology (Cain et al., 2009). To this end, we simulated a 2 min period of the dynamic behavior of the cardiovascular system under nominal conditions. This time duration was chosen to allow the simulated hemodynamic behavior to reach steady-state, similar to prior simulated cardiovascular behaviors generated by the original model (Ursino, 1998).

Next, we used the simulated hemodynamic behaviors to calculate quantitative metrics such as the averages of end diastolic pressure (EDP) and volume (EDV), end systolic pressure (ESP) and volume (ESV), left ventricular pressure, left ventricular flow rates, ejection fraction (EF, fraction of blood pumped out of the ventricle after systole), mean arterial pressure (MAP, a weighted average of systolic and diastolic pressure), and CO (total volume of blood pumped by the heart per unit time). Average values were calculated using mean hemodynamic and cardiovascular behaviors based on the final simulated 15 s, a sufficient amount of time to measure heart rate accurately (Kobayashi, 2013). These quantitative metrics of the simulated hemodynamic behaviors were then compared to values reported in the literature to evaluate how well the extended model was able to reproduce cardiovascular system behavior corresponding to healthy (Table 1, Cain et al., 2009, Supplementary Table S24) or systolic heart failure patients (Paulus et al., 2007; Klein and De Ferrari, 2010; Hung et al., 2010; Schwartzenberg et al., 2012). Sets of parameter estimates that resulted in simulation outputs corresponding to healthy or heart failure patients were then identified as baseline model parameters for healthy- or heart failure-conditions, respectively.

**TABLE 1:** Hemodynamic metrics for adult females (64 kg).

Parameter	Predicted values	Reported values		Units
SBP	121.20	123	(11)	mmHg
DBP	73.22	72	(8)	mmHg
EDV	128.30	118	(19)	mL
ESV	54.60	43	(11)	mL
EDP	13.20	9	(3)	mmHg
ESP	99.45	120	(20)	mmHg
EF	0.57	0.64	(0.08)	–
CO	82.88	85	(21)	mL/s
SV	82.90	75	(15)	mL/beat

*Values in parenthesis represent one standard deviation as reported (Cain et al., 2009). Abbreviations: systolic blood pressure (SBP), diastolic blood pressure (DBP), end diastolic volume (EDV), end systolic volume (ESV), end diastolic pressure (EDP), end systolic pressure (ESP), ejection fraction (EF), cardiac output (CO), and stroke volume (SV).*

### Compensatory Effects of Brainstem Neuronal Adaptation

To determine if brainstem neurons could adapt to compensate for the sustained physiological perturbations in the heart due to systolic heart failure, we assessed how changing parameter values from the baseline model parameters representing systolic heart failure affected simulated cardiovascular behavior. Specifically, we modified only parameters for transfer functions describing the dynamic behavior of brainstem neurons to represent neuronal adaptation. Modified parameter values were randomly selected within the constrained parameter space, as described previously in the brainstem parameter value selection process. We then ran simulations using these modified parameter values to determine what effect neuronal adaptations had on overall hemodynamic and cardiovascular behavior. We repeated this process 5000 times, selecting parameter value sets via Sobol sampling (Bratley and Fox, 1988; Hong and Hickernell, 2003; Joe and Kuo, 2003). Using this process, we conducted three sets of 5000 simulations, modifying different groups of brainstem neurons including: (1) all brainstem neurons, (2) NTS neuronal subtypes only, and (3) NA and DMV neuronal populations only to determine the extent to which brainstem neuronal adaptation is required to compensate for the physiological changes resulting from HFrEF.

### PAWN Global Sensitivity Analysis

Understanding the relative contribution of each cardiac- and neuronal-related parameter to overall behavior of the cardiovascular system would enable one to prioritize these parameters for further investigation, i.e., factor prioritization (Saltelli et al., 2008). To determine the relative contribution of each model parameter to variability in hemodynamic and cardiovascular behaviors under healthy, nominal conditions we performed a sensitivity analysis. Generally, sensitivity analysis is a multi-step method in which simulations using nominal parameter values are performed to determine the predicted system response to some specified perturbation. Parameter values are then changed systematically from their respective nominal values and simulations are repeated. Next, differences between the predicted responses that corresponding to the nominal parameter set and changed parameter set are analyzed to determine how sensitive these responses are to parametric changes. Two main types of sensitivity analyses typically used in the analysis of complex biological models are local- and global-sensitivity analysis, which are defined by the extent to which parameter values being examined are varied from their respective nominal values.

In this work, we performed a global sensitivity analysis because our investigation into the effects of neuronal adaptation showed that large changes in neuronal parameters (approximately three times the baseline systolic heart failure value in some instances) were required to shift hemodynamic and cardiovascular behaviors associated with systolic heart failure to nominal ranges. We applied a density-based sensitivity analysis approach known as PAWN (Pianosi and Wagener, 2015) to determine sensitivity indices for cardiac- and neuronal-related parameters with respect to four key metrics of cardiovascular behavior (HR, CO, MAP, and EF, Figure 6). We applied a density-based approach because the nature of the distribution of these metrics (e.g., unimodal, bimodal, etc.) was unclear and density-based approaches are robust to the shape of the model output distribution. Briefly, the PAWN methodology uses cumulative density functions (CDF) to characterize the model output of interest and determines output sensitivity to a parameter input of interest. Two sets simulations are used to determine the unconditional CDF and conditional CDF. The unconditional CDF is derived from a series of simulations where all inputs are varied simultaneously. The conditional CDF is derived from a separate set of simulations where all parameter inputs are varied except the parameter of interest, which is kept constant. The sensitivity index (*T*_*i*_) of the parameter of interest is then determined by comparing the distance between the unconditional and conditional CDF, defined by the Kolmogorov-Smirnoff (KS) statistic. Because the KS statistic is dependent upon the value at which the parameter of interest is kept constant, the PAWN method determines a KS statistic across multiple values for the parameter of interest, which span a pre-defined range of interest. Since multiple KS statistics are generated from this approach, a summary statistic (e.g., mean) characterizing the distribution of KS statistics generated from comparing the CDFs is used to define *T*_*i*_. The result is a sensitivity index for a parameter that ranges between 0 and 1, where larger values of *T*_*i*_, indicate greater parameter influence on the output. If *T*_*i*_ = 0, then the input, i.e., parameter of interest has no influence on the output (Pianosi and Wagener, 2015). In this analysis, we performed the default number of simulations defined in the PAWN methodology to determine the unconditional and conditional CDFs, respectively, from which the KS statistic was determined. The mean KS statistic was used to define the PAWN sensitivity index. In addition, the PAWN method also includes a bootstrapping test to determine a confidence interval for each sensitivity index. Here, we performed 1000 random samplings (with replacement) of the conditional CDFs (to compare against the unconditional CDF) for each bootstrapping test, which was performed for each parameter of interest.

### Simulating Exercise Response to Evaluate Contributions of Neuronal Adaptation in Healthy and Exercise Intolerant Phenotypes

We performed simulations using the “exercise-response” model to demonstrate the model’s ability to reproduce hemodynamic and cardiovascular behaviors consistent with reported cardiovascular system responses in healthy adults (Pawelczyk et al., 1992; Magosso et al., 2002). A 2 min period was also used to allow the simulated hemodynamic behavior to reach steady-state under exercise conditions. HR, CO, MAP, and systemic vascular resistance (SVR) were used to evaluate the predicted response of healthy patients to exercise. These metrics were determined based on the last 15 s of the 2 min simulation period.

To evaluate what systemic changes contribute to exercise intolerance, we used parameter values used to simulate cardiovascular and hemodynamic behaviors of HFrEF patients (i.e., modified cardiac parameters representing the damaged heart post systolic heart failure) in the “exercise-response” model. We then randomly selected different values within a ± 5-fold range from the nominal values for brainstem-associated parameters via Sobol sampling (Bratley and Fox, 1988; Hong and Hickernell, 2003; Joe and Kuo, 2003) to represent neuronal adaptation and performed a corresponding simulation. We repeated this process 5000 times to thoroughly examine the parameter space for each of the brainstem-associated parameters. Quantitative metrics including HR, CO, and MAP were used to compare model performance to reported exercise response behavior of HFrEF patients (Dhakal et al., 2015).

## Results

### Extended Model Reproduces Physiological Conditions of Healthy and HFrEF Patients

From the parameter estimation efforts, we identified baseline parameter values to simulate healthy and HFrEF conditions. A representative example of simulated pressure and volume outputs for both healthy and HFrEF conditions are illustrated in a P-V diagram with a subset of physiological outputs are tabulated in Figure 3. Metrics calculated from the simulated hemodynamic and cardiovascular behavior for both healthy and HFrEF conditions were well within reported ranges (Paulus et al., 2007; Cain et al., 2009; Klein and De Ferrari, 2010; Hung et al., 2010; Schwartzenberg et al., 2012), supporting the notion that the extended model provides a fair representation of short-term cardiovascular system dynamics in healthy and diseased states.

### Neuronal Adaptation Across Brainstem Neuronal Populations Provides Short-Term Compensation for Systolic Heart Failure

A comparison of the predicted hemodynamic and cardiovascular behaviors to the healthy cardiovascular characteristics (Table 1) revealed that only a few parameter sets were able to compensate adequately for impaired cardiac function. That is, only a few parameter sets representing neuronal adaptation led to a change in parasympathetic activity that would return cardiovascular behaviors and metrics quantifying these behaviors back to nominal levels. Interestingly, these parameters resulted in behaviors very similar to one another. Consequently, only one representative example of is shown in Figure 3.

We found that all of the adapted neuronal states that were successful in compensating for the physiologically perturbed cardiac state due to systolic heart failure led to decreases in vagal activity. For example, decreased sensitivity of the NA neuronal group to input firing frequency (rightward shift in the input-output relationship, Figure 3B) resulted in a constant, low vagal firing frequency that decreased heart rate (Figure 3C). In addition, decreases in firing frequency input to the transfer function describing the dynamic responses of the NA_contractility_ and decreased sensitivity to input firing frequencies of the DMV neuronal group resulted in decreased vagal activity that would reduce ventricular elastance. These changes caused an overall increase in elastance and a subsequent increase in CO, which is reduced in systolic heart failure patients with HFrEF.

Having demonstrated via simulation that neuronal adaptation can compensate for physiological changes resulting from systolic heart failure, we then sought to determine the extent to which particular subsets of neuronal subtypes or subpopulations contribute to the previously demonstrated compensatory effect (Figure 3). As the NTS regulates blood pressure set point and is central to baroreflex regulation (Rogers et al., 1993, 1996; Duan et al., 2009; Park et al., 2014), we examined the NTS and the neuronal subtypes therein separately from the NA and DMV, which have their own distinct and critical roles in regulating vagal outflow as well (Rinaman et al., 1989; Coote, 2013).

### Neuronal Adaptation in the NTS Cannot Provide Short-Term Compensation for Systolic Heart Failure

**FIGURE 4:**
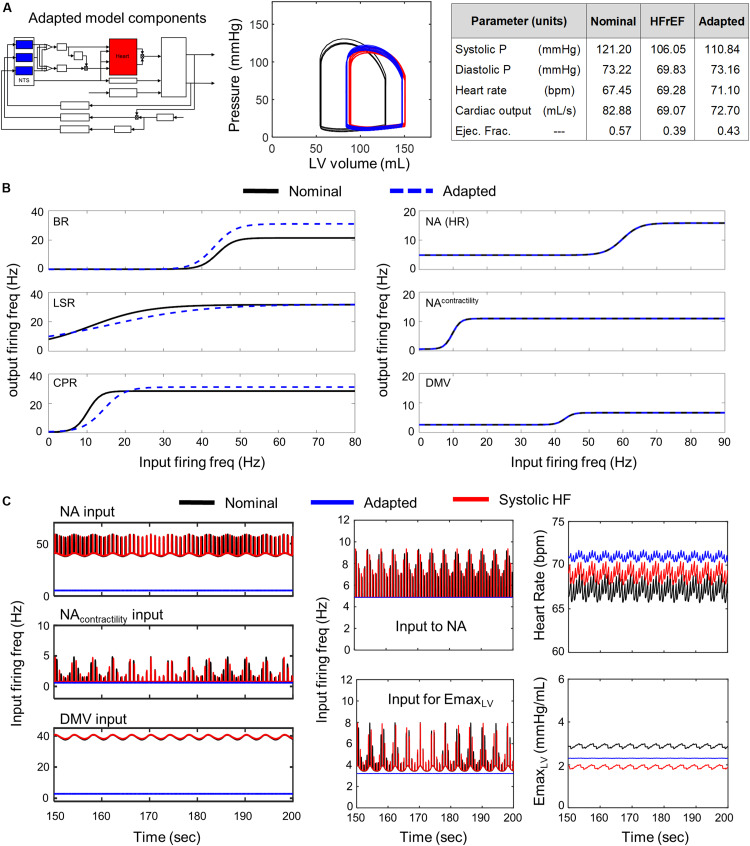
Effects of adaptation in NTS neuronal subtypes in HFrEF condition. Identical color schemes used in Figure 3 applies. **(A)** *Left pane*l – Schematic highlighting adjusted model components. *Middle panel* – simulated pressure-volume curve for baseline, heart failure, and adapted conditions. *Right panel* – A table summarizing cardiovascular outputs simulated by the model. **(B)** *Left panel* – sigmoidal curves representing input-output functions characterizing BR-, LSR-, and CPR-neuronal subtypes. Baseline and adapted input-output functions are plotted. *Right panel* – input-output functions characterizing NA-, NA_contractility_, and DMV-neuronal groups. Baseline and adapted input-output functions are plotted. **(C)** *Left panel* – Plots show input signal characteristics in brainstem due to adaptations occurring in NTS neuronal subtypes only over the last 50 s of a 200 s simulation for nominal, diseased, and adapted states. *Left top subpanel*: input ff signal received by NA neuronal population that primarily affects heart rate. *Left middle panel*: input ff signal to the NA neuronal population that primarily affects contractility. *Left bottom panel*: input ff signal to the DMV neuronal population that affects contractility. *Middle top panel*: simulated vagal activity modulating heart rate. This ff represents the sum of the output signals generated by the NA_contractility_ and DMV neuronal populations. *Middle bottom*: vagal activity that modulates ventricular elastance. *Right top panel*: resulting heart rate. *Right bottom panel*: resulting elastance of the left ventricle.

Of the 5000 different NTS-specific parameter sets tested, no parameter sets produced results where neuronal adaptation was able to compensate for impaired cardiac function and return cardiovascular system behavior to within nominal ranges. We observed only marginal improvements in the dynamic behavior of the cardiovascular system and in the quantitative metrics characterizing the predicted behavior. Changes to cardiovascular behavior due to NTS-specific adaptation included a slight decrease in EDV/ESV and a marginal increase in ejection fraction from 0.390 to 0.416, which remains below the lower limit of a normal ejection fraction (Figure 4). In the representative example shown in Figure 4, firing frequency outputs generated by adapted NTS neuronal subtypes were much lower than those predicted under nominal and diseased states. Consequently, the firing frequency output was too low to trigger a change in the NA and DMV output that would differ from those associated with systolic heart failure (Figure 4C middle subpanels). The result is a ventricular elastance response that is insufficient to increase cardiac output back to nominal levels (Figure 4C rightmost subpanels).

### Neuronal Adaptation in the NA and DMV Compensates Partially for Systolic Heart Failure

**FIGURE 5:**
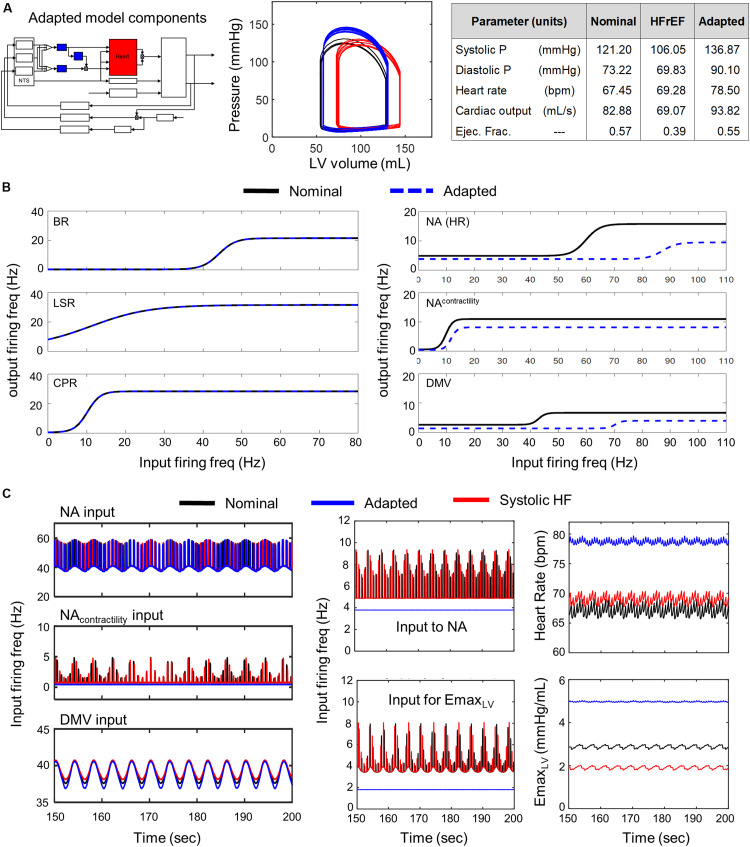
Effects of adaptation in NA and DMV neuronal subtypes in HFrEF condition. Identical color schemes used in Figure 3 applies. **(A)** *Left panel* – Schematic highlighting adjusted model components. *Middle panel* – simulated pressure-volume curve for baseline, heart failure, and adapted conditions. *Right panel* – a table summarizing physiological outputs simulated by model. **(B)** *Left panel* –sigmoidal curves representing input-output functions characterizing BR-, LSR-, and CPR-neuronal subtypes. Baseline and adapted input-output functions are plotted. *Right panel* – input-output functions characterizing NA-, NA_contractility_-, and DMV-neuronal groups. Baseline and adapted input-output functions are plotted. **(C)** *Left panel* – input signal characteristics in brainstem due to adaptations occurring in NTS neuronal subtypes only. Plots show ff outputs over the last 50 s of a 200 s simulation for nominal, diseased, and adapted states. *Left top subpanel*: input ff signal received by the NA neuronal population that primarily affects heart rate. *Left middle panel*: input ff signal to the NA neuronal population that primarily affects contractility. *Left bottom panel*: input ff signal to the DMV neuronal population that affects contractility. *Middle top panel*: vagal activity that modulates heart rate. This ff represents the sum of the output signals generated by brainstem neuronal populations represented by the NA_contractility_ and DMV transfer functions. *Middle bottom*: vagal activity modulating ventricular elastance. *Right top panel*: resulting heart rate. *Right bottom panel*: resulting elastance of the left ventricle.

Conversely, simulations involving modified parameter values of the transfer functions modeling the dynamic behavior of the NA, NA_contractility_, and DMV neuronal groups revealed that several aspects of cardiovascular system behavior could be restored to nominal levels (Figure 5 P-V loop). Simulations using parameter estimates representing adaptation of the NA, NA_contractility_, and DMV resulted in a CO (92.82 mL/s) and ejection fraction (0.55) well within nominal ranges. However, such compensation comes at a physiological cost, namely a concurrent increase in systolic and diastolic blood pressures nearing hypertensive levels (Figure 5A). The compensated hemodynamic behavior simulated by the extended model was due in part to a decrease in vagal activity, attributable to decreases in the sensitivity to firing frequency input and the minimal firing frequency of the NA, NA_contractility_, and DMV subpopulations (Figure 5C). As a result, the firing frequency output generated by these subpopulations (i.e., vagal activity) is lower than that generated by non-adapted neuronal subpopulations under systolic heart failure conditions (Figure 5C middle subpanels). Ultimately, the decrease in vagal activity regulating heart rate and elastance led to an approximate 13% rise in CO from nominal conditions, which contributed to the near-hypertensive systolic and diastolic blood pressures (Figure 5A).

**FIGURE 6:**
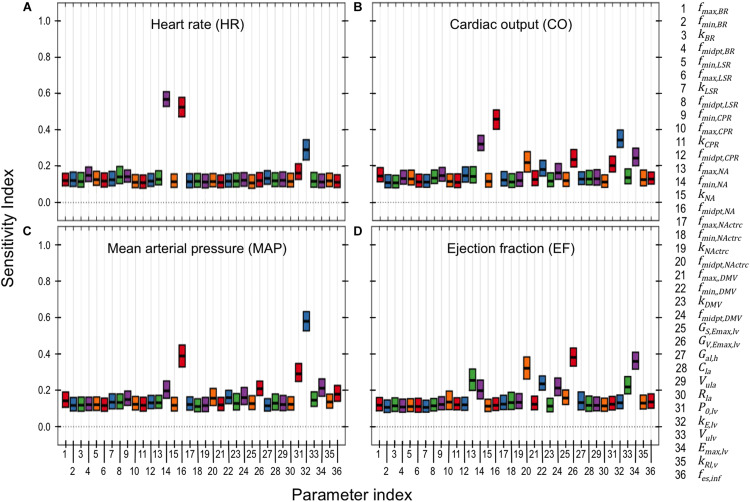
PAWN global sensitivity analysis. Global sensitivity indices were determined for neuronal and cardiac parameters using the PAWN sensitivity method (Pianosi and Wagener, 2015). These indices range between 0 and 1, with 0 indicating that the model parameter has no impact on the respective cardiovascular output (e.g., heart rate), and 1 indicating that the parameter of interest has a major influence on the respective cardiovascular output. PAWN sensitivity indices for neuronal and cardiac parameters were based on a ± 5-fold range from nominal parameter values, and were calculated with respect to **(A)** heart rate (HR), **(B)** cardiac output (CO), **(C)** mean arterial pressure (MAP), and **(D)** ejection fraction (EF). Each boxplot represents the 95% confidence interval of the sensitivity index based on bootstrapping tests that involved 1000 iterations of random sampling of simulated HR, CO, MAP, and EF used to determine the conditional CDFs.

This two-part simulation analysis of neuronal adaptation occurring in the NTS or NA/DMV has shown that adaptation of multiple neuronal subtypes and groups in the brainstem must occur in order to maintain cardiovascular homeostasis under a state of impaired cardiac function due to systolic heart failure. Moreover, the limited number of parameter sets that resulted in compensated cardiovascular behavior under systolic heart failure conditions (Figure 3) suggests that a limited regime of neuronal subtype states in the brainstem can produce the desired cardiovascular behavior necessary to maintain homeostasis under such conditions.

### PAWN Global Sensitivity Analysis of Cardiac- and Neuronal-Parameters

While the simulation analysis of subpopulation-specific adaptations indicated that cardiac- and neuronal-related parameters affect hemodynamic and cardiovascular behaviors, the extent to which each of these individual parameters contribute to variation in these behaviors under healthy conditions remained unclear.

Results from the PAWN sensitivity analysis indicates that HR, CO, MAP, and EF are relatively sensitive to only a few (2–4) parameters out of the 36 neuronal- and cardiac-related parameters analyzed (Figure 6). These parameters include *f*_*min,NA*_, *f*_*midpt,NA*_, *f*_*midpt,NActrc*_, (neuronal-related parameters), *k*_*E,lv*_, *P_0__,lv_*, *E*_*max,lv*_, (cardiac-related parameters), and *G*_*V,Emax,lv*_ (autonomic-related parameter). HR was nearly equally sensitive to *f*_*min,NA*_, *f*_*midpt,NA*_. In the context of the extended model, HR is determined primarily by firing frequency generated by the NA subpopulation. Thus it is reasonable to expect that *f*_*min,NA*_, the basal firing frequency output, and *f*_*midpt,NA*_, which influences how sensitive the NA subpopulation is to input signals, would have major contributions to HR variation. Interestingly, *f*_*min,NA*_ and *f*_*midpt,NA*_ have larger sensitivity indices than *k*_*E,lv*_, a parameter which helps define the pressure/volume relationship during the diastolic phase of the cardiac cycle. This may suggest a relative importance of neuronal activity over cardiac-associated parameters in modulating HR. Conversely, *k*_*E,lv*_ has the largest contribution to variation in CO. As cardiac blood flow depends on the pressure/volume relationship at diastole, it is not surprising that *k*_*E,lv*_ affects CO to this extent. Similarly, *k*_*E,lv*_ has the largest contribution to MAP variation, which is also highly dependent upon ventricular pressure during the cardiac cycle. Finally, EF is most sensitive to *f*_*midpt,NActrc*_, *G*_*V,Emax,lv*_, and *E*_*max,lv*_, which are parameters that indicate the influence vagal activity has on ventricular elastance. These three parameters have similar sensitivity index values, ranging between 0.3 − 0.4, which we interpret as evidence that support the importance of vagal regulation, particularly because EF is a key feature used to define HFrEF.

### Role of Coordinated Regulation in Dealing With Acute Stress (Exercise Intolerance)

Based on the previous simulation analysis in which we showed that neuronal adaptation in the brainstem could provide short-term compensation for systolic heart failure, we were motivated to investigate how maladaptation and dysregulation of cardiac functions may contribute to other clinical phenotypes associated with systolic heart failure. Exercise intolerance, a common clinical phenotype associated with systolic heart failure, has been the subject of several clinical and modeling studies – many of which have focused on understanding how physical changes in the heart, peripheral vasculature, and metabolic activity post systolic heart failure contribute to exercise intolerance. Since neuronal activity is difficult to measure directly in patients, changes in neurohormonal activity and other circulating biomarkers such as AngII are used as indirect measures of sympathetic activity in patients. The extended model developed here provides an alternative mathematical tool that can be used to investigate how autonomic dysregulation contributes to exercise intolerance.

**FIGURE 7:**
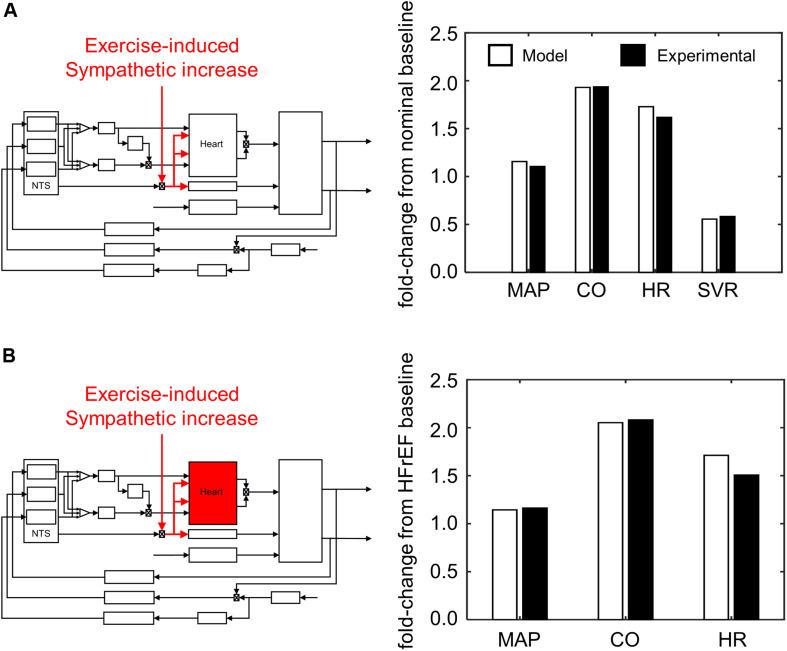
Simulating exercise-stress response. **(A)** Schematic illustrating effect of exercise on sympathetic tone and its impact on the overall cardiovascular system. Values of mean arterial pressure (MAP), cardiac output (CO), heart rate (HR), and systemic vascular resistance (SVR) are calculated from hemodynamic and cardiovascular behaviors simulated by the model under healthy conditions, and subsequently compared to experimental measures of healthy patients (Pawelczyk et al., 1992). **(B)** A parallel schematic comparing experimental and simulated results of exercise stress response (V_O__2_ 1L/min) under HFrEF conditions. All ratios are expressed as fold-change relative to values measured at rest of HFrEF patients (Dhakal et al., 2015). Because SVR was not measured by Dhakal et al., it was not included as part of the criteria to evaluate parameter estimates for the HFrEF condition.

From our parameter estimation efforts using the “exercise response” model, we were able to reproduce cardiovascular and hemodynamic behaviors of healthy and HFrEF patients undergoing an exercise-induced stress (Figure 7). Assessing the results from our simulation analysis, we found that only four parameter sets (Supplementary Table S23) of the 5000 tested resulted in simulated behavior similar to those of exercise intolerant HFrEF patients (Dhakal et al., 2015). In these four cases, the parameter sets represented adaptive responses that caused a decrease in sensitivity to input firing frequencies of the NA, NA_contractility_, and DMV neuronal groups (Supplementary Figure S2). This decrease in sensitivity caused an overall decrease in vagal activity regulating the heart and a subsequent increase in HR and ventricular elastance to meet the demands of an increased metabolic load caused by the onset of exercise.

**FIGURE 8:**
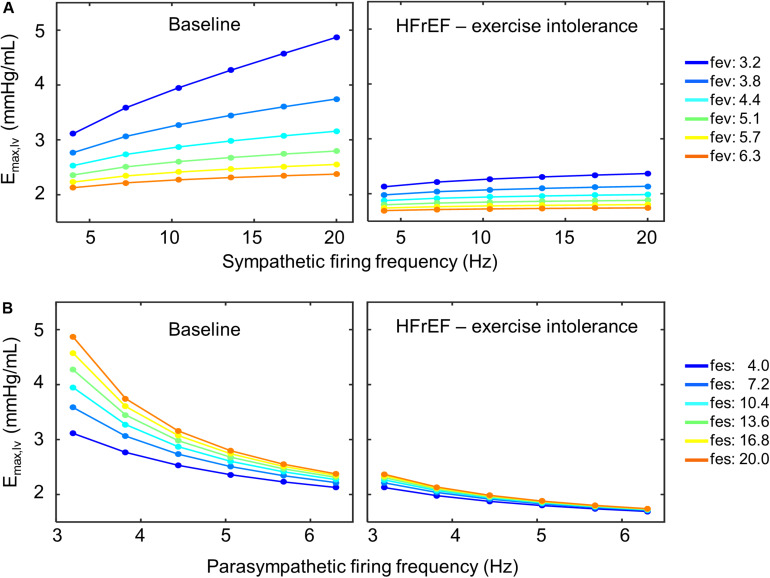
Loss of accentuated antagonism and exercise intolerance. **(A)** Top row shows the effect of accentuated antagonism on maximum left ventricular elastance (*E*_*max,lv*_) in both healthy (*right*) and HFrEF (*left*) simulated conditions. Here, ventricular elastance increases as sympathetic ff increases. As vagal activity (*f*_*ev*_) increases, so does its inhibitory effect on ventricular elastance. **(B)** Larger decreases in ventricular elastance occur under simultaneous sympathetic activity (*f*_*es*_). Accentuated antagonism is lost, however, in HFrEF conditions.

Closer examination of these parameter sets revealed that two of the four sets included a decrease in sympathetic gain (*G*_*s,Emax*_) and increase in parasympathetic gain (*G*_*v,Emax*_) to ventricular elastance, indicating a change in the extent to which ventricular elastance responds to autonomic signals. To understand the implications of the changes in *G*_*s,Emax*_ and *G*_*v,Emax*_, we examined the elastance response over a range of sympathetic and parasympathetic activity levels. Under nominal conditions, the accentuated antagonistic relationship between the sympathetic and parasympathetic branches leads to larger changes in elastance when both branches simultaneously modulate the effector function than if either branch were acting in isolation. However, this accentuated antagonistic effect on elastance was nearly abolished when *G*_*s,Emax*_ and *G*_*v,Emax*_ decreased and increased, respectively (Figure 8). As a result, elastance varied marginally across the range of sympathetic and parasympathetic activities tested.

**FIGURE 9:**
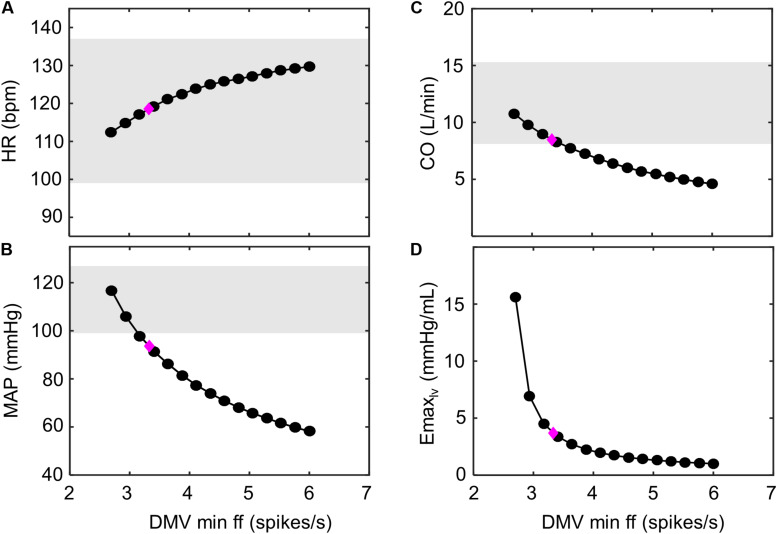
Improving hemodynamic response to exercise-stress. Decreasing the minimum ff threshold of the DMV neuronal group has a monotonic effect on multiple hemodynamic parameters. This is observed as **(A)** an increase in heart rate (HR), as **(B)** a decrease in mean arterial pressure (MAP), as **(C)** a decrease in cardiac output (CO), and **(D)** a decrease in left ventricular elastance (*E*_*max,lv*_). The magenta diamond indicates the hemodynamic outputs associated with the model parameters simulating exercise response (V_O__2_ ∼ 1 L/min) of HFrEF patients shown (Figure 7B). Gray regions represent experimentally measured ranges of hemodynamic behavior of healthy patients (V_O__2_ ∼1 L/min; Andersen et al., 2015). Because elastance was not previously measured by Andersen et al. (2015), experimental ranges were not include in **(D)**.

We subsequently explored several potential parameter modifications that would provide short-term improvement in cardiovascular performance. Given the loss of accentuated antagonism affecting elastance, we suspected that decreasing *G_*v,Ema*__*x*_* to restore the ratio between *G*_*s,Emax*_ and *G*_*v,Emax*_ to a value similar to the ratio found under nominal conditions would effectively reestablish the accentuated antagonistic effect. Indeed, our simulation analysis indicated that decreasing *G*_*v,Emax*_ would improve HR, MAP, and CO to nominal levels (Supplementary Figure S3). However, these results were only achievable using physiologically unrealistic values for ventricular contractility (>8.0 mmHg/mL) – values, which to the best of our knowledge, have never been observed experimentally or reported in the literature. Alternatively, decreasing the minimal firing rate parameter in the transfer function characterizing the dynamic response of the DMV neuronal group (*f*_*min,DMV*_) resulted in an increase in ventricular elastance and CO that were within the nominal range. Unfortunately, unrealistic ventricular elastance values (Figure 9) were required to produce the predicted CO levels. Results from the PAWN sensitivity analysis indicate that *f*_*midpt,NA*_, the firing frequency that leads to a half-maximal output of the NA subpopulation, would be the primary candidate to modulate in order to increase CO, as *f*_*midpt,NA*_ has the highest sensitivity index (Figure 6). Although increasing the *f*_*midpt,NA*_ would theoretically increase CO, this increase would consequently increase HR and MAP. A larger *f*_*midpt,NA*_ value would cause a rightward shift in the input sensitivity of the NA subpopulation, meaning that larger input firing frequencies would be required to trigger a change in NA output. The result would be a subsequent decrease in vagal activity and an increase in HR and MAP. These theoretical consequences are further supported by results from the PAWN sensitivity analysis, which indicates *f*_*midpt,NA*_ has the largest or 2^nd^ largest contribution to HR and MAP variation, respectively, among the model parameters analyzed.

## Discussion

In this study, we investigated the functional relevance of adaptation occurring within distinct neuronal subtypes and neuronal subpopulations of the brainstem in the context of autonomic regulation of cardiovascular function. By developing a closed-loop model of short-term baroreflex regulation of cardiovascular functions, we investigated how neuronal adaptation affects parasympathetic activity and contributes to the dynamic hemodynamic and cardiovascular behaviors associated with HFrEF and exercise intolerance. Results from our simulation analysis suggest that brainstem neuronal adaptation, effected by changes to parameters in the transfer function models representing the dynamic behavior of brainstem neurons, can compensate for impaired cardiac function of the heart, and restore cardiovascular performance to within nominal ranges. Such compensation was due to increased ventricular elastance mediated by a decrease in vagal activity. Recall that the extended model characterizes cardiovascular function on a short time-scale (order of minutes), by not considering neurohormonal or metabolic activity that contributes to sustained behavior observed over longer time-scales (hours to days). Consequently, we interpret such indicated decreased vagal activity to be a short-term compensatory response to systolic heart failure-induced impaired cardiac function. Interestingly, when simulating the cardiovascular system behavior under systolic heart failure conditions in which adaptation occurred in all neuronal subtypes and groups (Figure 3), ventricular elastance (∼5 mmHg/mL), CO (86 mL/s), and EF (0.59) aligned well with those of patients suffering from heart failure with *preserved* ejection fraction (HFpEF) (Kawaguchi et al., 2003). These results, taken in context with our results that reproduced cardiovascular behavior of HFrEF patients (Figure 3), suggest that the functional state of brainstem neurons determine, in part, the clinical phenotype a systolic heart failure patient may exhibit, i.e., HFrEF or HFpEF. Further simulation analyses exploring neuronal adaptation in the context of HFpEF, where diastolic dysfunction as opposed to systolic dysfunction is a contributing factor, are required and will be considered in future work.

Given the contribution of neuronal adaptation in defining the dynamic cardiovascular behavior of patients suffering from HFrEF, we examined the role of neuronal adaptation in exercise intolerance. We modified the extended model to account for changes associated with exercise onset by: (1) adding separate vascular compartments for resting and active muscles, (2) including a lower body muscle pump to simulate the periodic contraction of the legs, (3) adding an exercise-specific respiratory responses and consequent changes to thoracic pressure, and (4) adding a positive offset to sympathetic activity during exercise. Simulation analysis revealed that the adaptive responses of brainstem neurons were sufficient to produce simulation results that matched experimentally measured cardiovascular responses of healthy patients challenged to an acute bout of exercise. However, to reproduce results matching the cardiovascular performance of exercise-intolerant HFrEF patients, new parameter values for the *G*_*s,Emax*_ and *G*_*v,Emax*_, the constant gains that quantify the relationship between sympathetic and parasympathetic activity to elastance were also required. In several cases, the new estimates of *G*_*s,Emax*_ and *G*_*v,Emax*_ led to a loss in the accentuated antagonistic relationship between the sympathetic and parasympathetic branches regulating ventricular elastance (Figure 8). Consequently, re-adjusting the sympathetic and parasympathetic gains on ventricular elastance such that the *G*_*s,Emax*_:*G*_*v,Emax*_ ratio was similar to the ratio associated with nominal conditions improved the cardiovascular performance of HFrEF patients; predicted HR, MAP, and CO levels were similar to those of healthy patients observed at V_O__2_ of 1L/min. These results further support the importance of autonomic balance in maintaining healthy cardiovascular behaviors.

Our analyses indicate that to compensate adequately for impaired cardiac function, neuronal adaptation *must* occur in multiple neuronal populations. Under the impaired state due to systolic heart failure, adaptations occurring only in NTS neuronal subtypes were not sufficient to restore hemodynamic and cardiovascular behavior to within nominal ranges, nor could they improve cardiac output and ejection fraction to nominal levels. The necessity for adaptation to occur in the neuronal populations of the NA and DMV (Figure 5) is consistent with the key role that these nuclei are known to play in regulating cardiovascular function via vagal activity. The changes in parameters of the transfer functions modeling the dynamic behavior of the NA, NA_contractility_, and DMV neuronal groups required to return cardiovascular performance to nominal levels indicate that these neuronal populations may act as filters or synaptic gates, modulating the input signals generated by the neuronal subtypes in the NTS. This gating effect can provide a short-term compensatory effect, where suppressed vagal activity can lead to an overall increase in cardiac effector function activity like ventricular elastance. In the case of HFrEF, the cardiovascular system is predicted to behave within nominal ranges, albeit near the upper limits of the nominal, healthy levels (e.g., near hypertensive blood pressures were predicted). Conversely, a sustained gating effect maintained by neuronal populations in the NA and DMV would theoretically lead to a sustained decrease in vagal activity, which has been linked to poor long-term cardiovascular health in patients who have suffered from heart failure. In fact, reversing decreased vagal activity in HF patients has been the recent focus of multiple clinical trials to improve long-term cardiovascular health in heart failure patients (De Ferrari and Schwartz, 2011). It is plausible, therefore, that targeting molecular mechanisms that affect neuronal firing frequency within the NA and DMV may provide a means to increase vagal activity and improve the overall autonomic imbalance associated with heart disease.

### Model Limitations and Future Work

While this extended model represents, to the best of our knowledge, the most comprehensive quantitative characterization of multiple neuronal components involved in cardiovascular regulation, it has limitations worth noting. Only a few select afferent input-types were included in this model, even though the NTS receives a diverse array of afferent and higher-order inputs from other brain regions. For example, chemoreceptor afferents terminate in the NTS and play a major role in cardiovascular regulation. Although chemoreceptors have a larger influence in regulating pH and CO_2_ levels in the blood, they certainly affect cardiovascular and respiratory function. In addition, metabolic activity, which plays a large role in modulating oxygen consumption and cardiovascular response to exercise demands (Magosso and Ursino, 2002), was not included in this particular model. Furthermore, neuronal populations in the DMV generate spontaneous rhythmic firing patterns. These pacemaker neurons play a critical role in modulating heart rate and respiration, both of which affect cardiovascular function as well (Jones et al., 1998; Coote, 2013). While these omissions limit the types of physiological conditions that can be explored with this model, they provide opportunities to improve the model so that it can be used to identify additional neuronal mechanisms of interest. Further, the contributions of autonomic (dys)regulation can be analyzed in different disease conditions. For example, resistant hypertension – a condition defined as the failure to achieve a blood pressure target despite the use of 3 or more antihypertensive agents; 1 of which is a diuretic (Calhoun et al., 2008), may be a plausible disease condition that can be analyzed. Of course, appropriate modifications to the model should be made to reflect typical patient characteristics such as obesity and left ventricular hypertrophy (Calhoun et al., 2008).

## Conclusion

We have extended prior models of baroreflex regulation of cardiovascular function by including brainstem neuronal components and functional connectivity among these components that regulate vagal activity, a critical element of cardiovascular homeostasis. This extended model provides a platform for examining the functional relevance of neuronal adaptation, as suggested by the single-neuron analysis of the NTS (Park et al., 2014). The adaptive capabilities of neuronal subtypes/subpopulations in the brainstem provide a mechanism through which the autonomic nervous system is able to modulate parasympathetic activity and regulate cardiac functions (e.g., ventricular elastance). Under impaired conditions like systolic heart failure, this adaptive capability helps to maintain certain hemodynamic and cardiovascular behaviors like blood pressure within normal, healthy ranges. As sustained inhibition of vagal activity is characteristic of systolic heart failure, it remains unclear if neuronal adaptations, alternative mechanisms, or a combination of the two cause the sustained suppression of vagal activity in heart failure patients. Further analysis is required to elucidate these mechanisms. Since modulating vagal activity to improve cardiovascular health in heart failure patients has been an active area of clinical trials, future investigations providing insights into potential mechanisms leading to a sustained inhibition of vagal activity would be greatly beneficial.

## Data Availability Statement

The raw data supporting the conclusions of this article will be made available by the authors, without undue reservation, to any qualified researcher.

## Author Contributions

JP conceptualized the research goals and aims, developed the extended model and methodology used for simulation analysis, performed formal analysis of simulation results, created figures, wrote, edited, and reviewed the manuscript. JG contributed to conceptualization, writing, and editing. BO contributed to conceptualization, analysis, writing, and editing. JS and RV contributed to conceptualization and editing.

## Conflict of Interest

The authors declare that the research was conducted in the absence of any commercial or financial relationships that could be construed as a potential conflict of interest.

## Supplementary Material

FIGURE S1 Open-loop and closed-loop modeling conditions for parameter selection. **(A)** Open-loop condition used to simulate heart rate and contractility for selecting parameters parameter of transfer functions representing the input-output behavior of the baroreceptor input-driven neuronal subtype, NA, NA_contractility_, and DMV subgroups. The blue-colored path and control blocks correspond to the flow of information in which systemic arterial pressure (*P*_*sa*_) is used to stimulate the aforementioned neuronal subtypes and subgroups and ultimately cause a change in heart rate and ventricular elastance from basal levels. The gray colored control blocks indicate sensory neurons whose transfer function parameters were known (Ursino, 1998). **(B)** Open-loop condition used for selection of parameters corresponding to the lung-stretch receptor input-driven neuronal subtype (blue-colored control block in NTS). Lung volume (*V*_*lung*_) was used as an input parameter to stimulate a change in heart rate and ventricular elastance from basal levels. The parameters for the NA, NA_contractility_, and DMV determined from part A were used (gray colored control blocks) during the parameter selection process. **(C)** Closed-loop condition used to simulate heart rate, stroke volume, and cardiac output to for selecting parameters for cardiopulmonary receptor input-driven neuronal subtype (blue-colored control block in NTS). Lower body negative pressure (LBNP) was used as input parameter, which affected systemic circulation, to cause a change in heart rate, stroke volume, and cardiac output. The same parameters for the transfer functions corresponding to the NA, NA_contractility_, and DMV determined in part A were used in closed-loop model simulations.

FIGURE S2 Representative simulated neuronal response to exercise stress in HFrEF condition. **(A)** *Left panel* – Plot of sigmoidal curves representing input-output functions characterizing BR-, LSR-, and CPR-neuronal subtypes. Baseline and adapted input-output functions are plotted. *Right panel* – plot of input-output functions characterizing the NA-, NA_contractility_-, and DMV-neuronal groups. Baseline and adapted input-output functions are plotted. **(B)** *Left panel* – Plots of input signal characteristics in brainstem due to adaptations occurring in NTS neuronal subtypes only. Plots show ff outputs over the last 50 s of a 200 s simulation for nominal, diseased, and adapted states. *Left top subpanel*: plot of input ff signal received over time by the NA neuronal population that primarily affects heart rate. *Left middle panel*: plot of input ff signal to the NA neuronal population that primarily affects contractility. *Left bottom panel*: plot of input ff signal to the DMV neuronal population that affects contractility. *Middle top panel*: firing frequency representing vagal tone that modulates heart rate. This ff represents the sum of the output signals generated by brainstem neuronal populations represented by the NA_contractility_ and DMV transfer functions. *Middle bottom*: vagal activity that modulates ventricular elastance. *Right top panel*: resulting heart rate. *Right bottom panel*: resulting contractility of the left ventricle.

FIGURE S3 Improving hemodynamic response to exercise-stress. Decreasing the minimum ff of the DMV neuronal group has a monotonic effect on multiple hemodynamic parameters. This is observed as **(A)** an increase in heart rate (HR), **(B)** a decrease in mean arterial pressure (MAP), **(C)** a decrease in cardiac output (CO), and **(D)** a decrease in left ventricular elastance (*E*_*max,lv*_). The magenta diamond indicates the hemodynamic outputs associated with the model parameters simulating exercise response (V_O2_ ∼1 L/min) of HFrEF patients shown (Figure 7B). Gray regions represent experimentally measured ranges of hemodynamic behavior of healthy patients (V_O2_ ∼1 L/min; Andersen et al., 2015). Because elastance was not measured by Andersen et al. (2015), experimental ranges were not included in **(D)**.

TABLE S1 Hemodynamic variables.

TABLE S2 Hemodynamic parameter values (vascular system).

TABLE S3 Hemodynamic parameters (left heart).

TABLE S4 Activation function parameters.

TABLE S5 Hemodynamic parameters (left heart).

TABLE S6 Afferent input parameters (baroreceptors).

TABLE S7 Afferent input parameters (cardiopulmonary receptors).

TABLE S8 Afferent firing frequency gains (to heart).

TABLE S9 Afferent firing frequency gains (to peripheral circulation).

TABLE S10 Afferent firing frequency gains (to unstressed volumes).

TABLE S11 Efferent sympathetic outflow parameters.

TABLE S12 Effector function regulation (gains).

TABLE S13 Effector function (time constants).

TABLE S14 Effector function (constants).

TABLE S15 Basal Respiration Parameters.

**TABLE S15B |** Exercise-associated Respiration Parameters.

TABLE S16 Neuronal subtype parameters (baroreceptor-input subtype).

TABLE S17 Neuronal subtype parameters (cardiopulmonary receptor-input subtype).

TABLE S18 Neuronal subtype parameters (lung-stretch receptor input subtype).

TABLE S19 Nucleus ambiguus neuronal population parameters (heart rate).

TABLE S20 Nucleus ambiguus neuronal population parameters (contractility).

TABLE S21 Dorsal motor nucleus neuronal population (contractility).

TABLE S22 NTS subtype gains to NA and DMV.

TABLE S23 Simulink model parameters.

TABLE S24 Summary of experiments that generated data used in parameter estimation.
