# Supplementary figures and images for: Investigating the Effects of Brainstem Neuronal Adaptation on Cardiovascular Homeostasis
Source: Front Neurosci. 2020 May 20;14:470. doi: 10.3389/fnins.2020.00470 (PMC7251082; doi:10.3389/fnins.2020.00470)

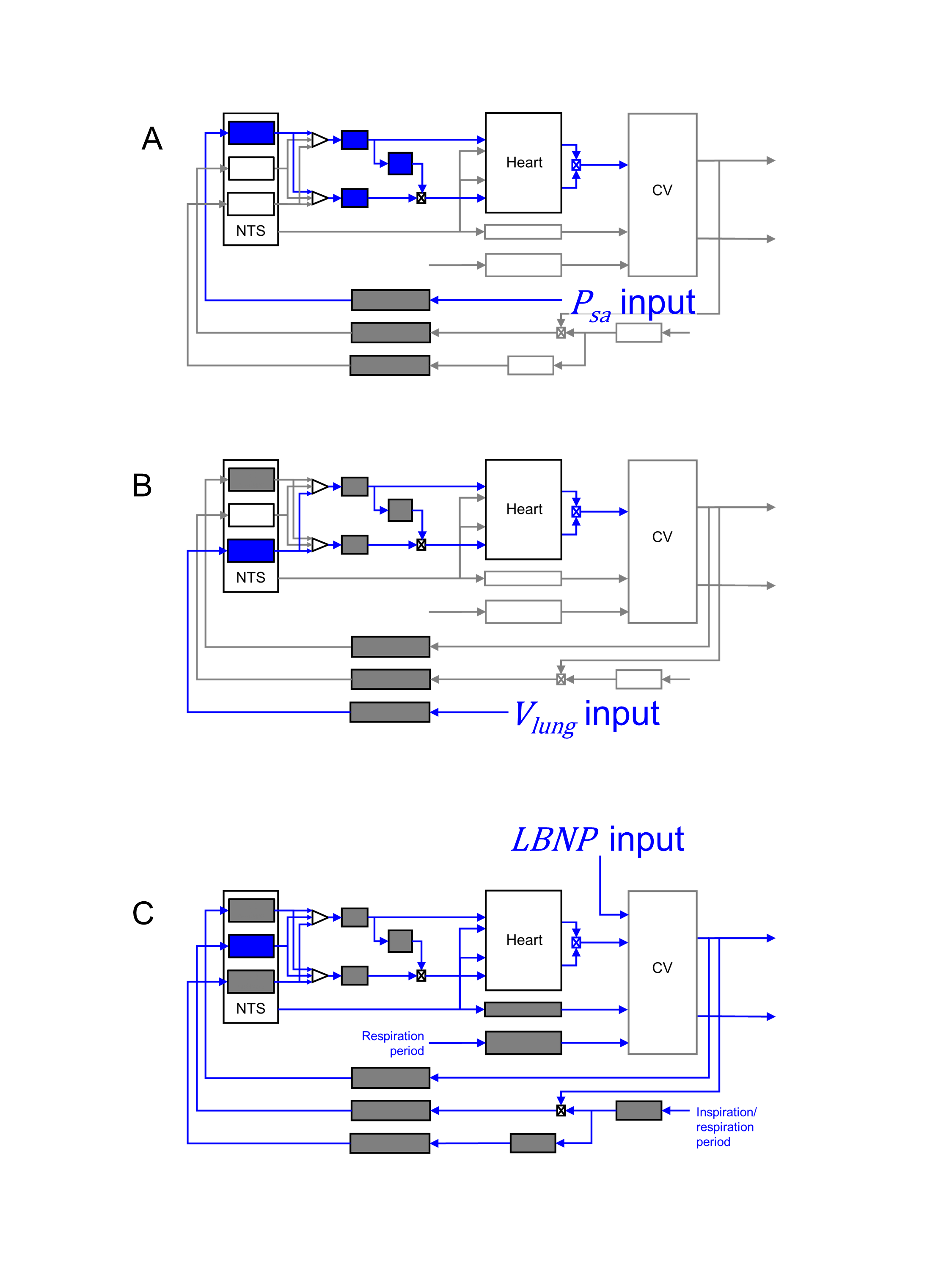

Supplement: FIGURE S1 — Open-loop and closed-loop modeling conditions for parameter selection. (A) Open-loop condition used to simulate heart rate and contractility for selecting parameters parameter of transfer functions representing the input-output behavior of the baroreceptor input-driven neuronal subtype, NA, NAcontractility, and DMV subgroups. The blue-colored path and control blocks correspond to the flow of information in which systemic arterial pressure (Psa) is used to stimulate the aforementioned neuronal subtypes and subgroups and ultimately cause a change in heart rate and ventricular elastance from basal levels. The gray colored control blocks indicate sensory neurons whose transfer function parameters were known (Ursino, 1998). (B) Open-loop condition used for selection of parameters corresponding to the lung-stretch receptor input-driven neuronal subtype (blue-colored control block in NTS). Lung volume (Vlung) was used as an input parameter to stimulate a change in heart rate and ventricular elastance from basal levels. The parameters for the NA, NAcontractility, and DMV determined from part A were used (gray colored control blocks) during the parameter selection process. (C) Closed-loop condition used to simulate heart rate, stroke volume, and cardiac output to for selecting parameters for cardiopulmonary receptor input-driven neuronal subtype (blue-colored control block in NTS). Lower body negative pressure (LBNP) was used as input parameter, which affected systemic circulation, to cause a change in heart rate, stroke volume, and cardiac output. The same parameters for the transfer functions corresponding to the NA, NAcontractility, and DMV determined in part A were used in closed-loop model simulations. [file Image_1.TIF]

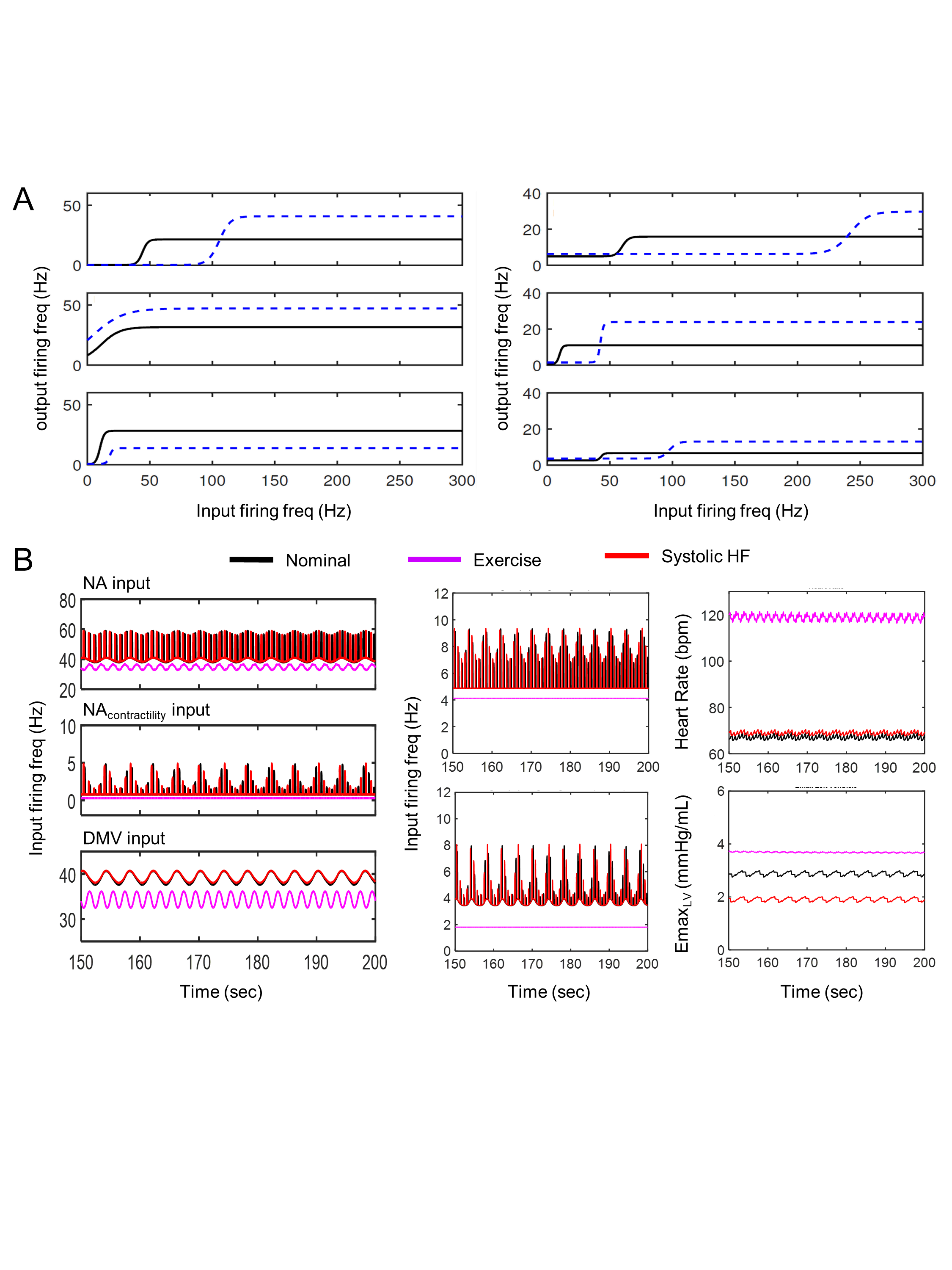

Supplement: FIGURE S2 — Representative simulated neuronal response to exercise stress in HFrEF condition. (A) Left panel – Plot of sigmoidal curves representing input-output functions characterizing BR-, LSR-, and CPR-neuronal subtypes. Baseline and adapted input-output functions are plotted. Right panel – plot of input-output functions characterizing the NA-, NAcontractility-, and DMV-neuronal groups. Baseline and adapted input-output functions are plotted. (B) Left panel – Plots of input signal characteristics in brainstem due to adaptations occurring in NTS neuronal subtypes only. Plots show ff outputs over the last 50 s of a 200 s simulation for nominal, diseased, and adapted states. Left top subpanel: plot of input ff signal received over time by the NA neuronal population that primarily affects heart rate. Left middle panel: plot of input ff signal to the NA neuronal population that primarily affects contractility. Left bottom panel: plot of input ff signal to the DMV neuronal population that affects contractility. Middle top panel: firing frequency representing vagal tone that modulates heart rate. This ff represents the sum of the output signals generated by brainstem neuronal populations represented by the NAcontractility and DMV transfer functions. Middle bottom: vagal activity that modulates ventricular elastance. Right top panel: resulting heart rate. Right bottom panel: resulting contractility of the left ventricle. [file Image_2.TIF]

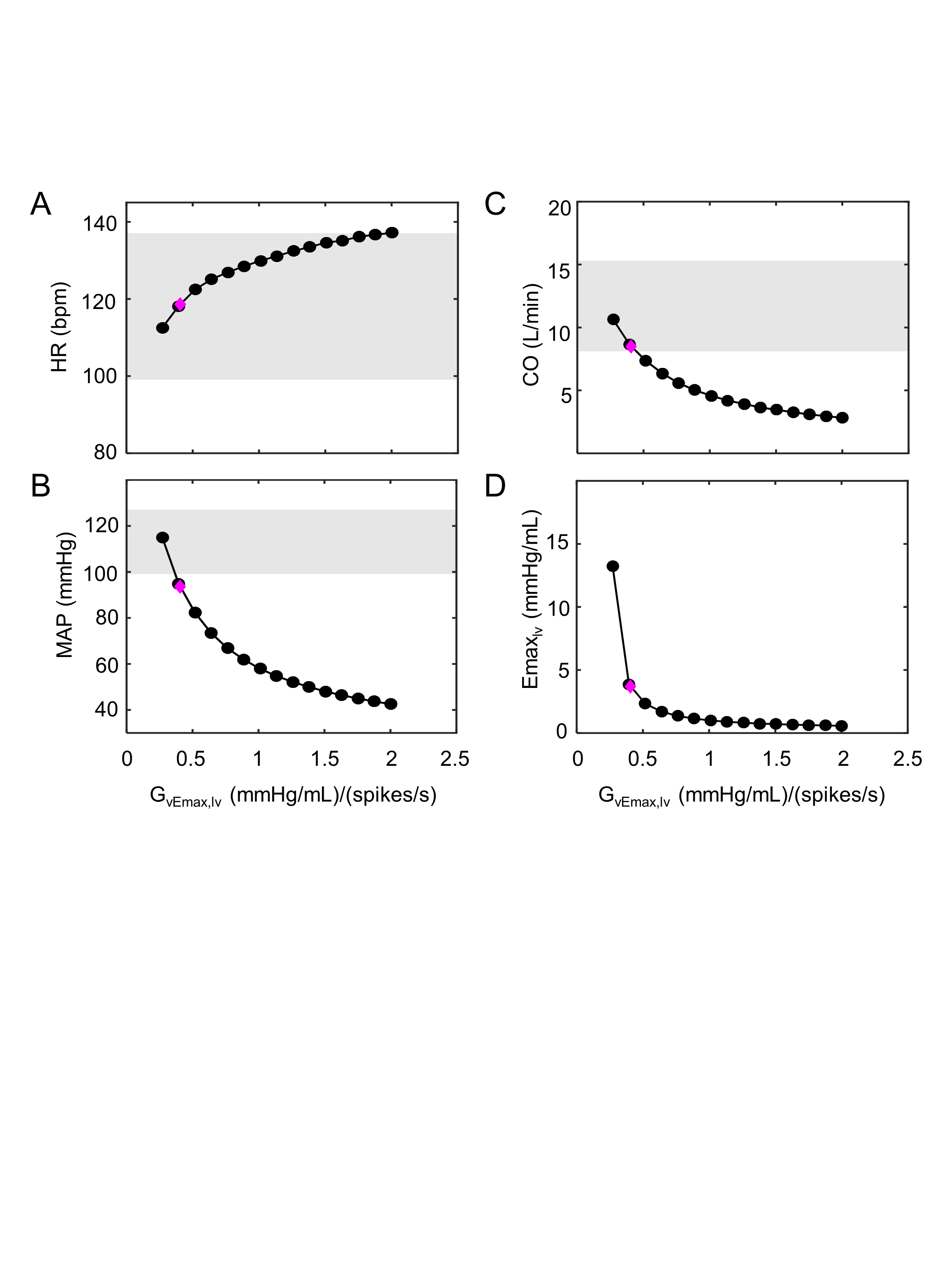

Supplement: FIGURE S3 — Improving hemodynamic response to exercise-stress. Decreasing the minimum ff of the DMV neuronal group has a monotonic effect on multiple hemodynamic parameters. This is observed as (A) an increase in heart rate (HR), (B) a decrease in mean arterial pressure (MAP), (C) a decrease in cardiac output (CO), and (D) a decrease in left ventricular elastance (Emax,lv). The magenta diamond indicates the hemodynamic outputs associated with the model parameters simulating exercise response (VO2 ∼1 L/min) of HFrEF patients shown (Figure 7B). Gray regions represent experimentally measured ranges of hemodynamic behavior of healthy patients (VO2 ∼1 L/min; Andersen et al., 2015). Because elastance was not measured by Andersen et al. (2015), experimental ranges were not included in (D). [file Image_3.TIF]
