# Supplementary material for: Investigating the Effects of Brainstem Neuronal Adaptation on Cardiovascular Homeostasis
Source: Front Neurosci. 2020 May 20;14:470. doi: 10.3389/fnins.2020.00470 (PMC7251082; doi:10.3389/fnins.2020.00470)
Supplement: TABLE S1 — Hemodynamic variables. [file Table_1.docx]

**Investigating the Effects of Brainstem Neuronal Adaptation on Cardiovascular Homeostasis**

**Supplemental Text**

**Brief Summary of Previous Select Models**

Because the baroreflex is a physiological control system, quantitative approaches from the field of control systems theory provide methodologies with which to systematically characterize and model this physiological reflex. Several efforts have focused on analyzing the sources of the oscillatory nature of blood pressure, which varies between a maximum systolic pressure and minimum diastolic pressure, and the stability of this oscillatory behavior. Burgess et al. (Burgess et al. 1997) developed a first-order linear feedback model describing the response of mean arterial pressure to changes in sympathetic drive. The model includes a proportional controller representing neural control mechanisms and a sensor or “feedback” gain. Using their model, Burgess et al. showed that high-frequency oscillations (~4 Hz) observed in the blood pressure of rats could be accounted for by the time-delays associated with the sympathetic response to blood pressure changes. One implication to this simple yet elegant model is that a strict relationship between the vasculature and sympathetic tone must exist to maintain stable blood pressure oscillations. Stimuli that would alter the gain in the feedback loop (e.g. altered baroreflex gain) would cause the system behavior to become either unstable (gain increase) or asymptotically stable (gain decrease) (Ben-Tal et al. 2014; Magosso & Ursino 2002). Therefore, in order to maintain stable oscillatory behavior, the linear feedback model suggests that continuous adaption must occur in the system.

Subsequent work by Ringwood and Malpas (2001) included nonlinear input-output relationships describing the central nervous system and vasculature components within the feedback model. By incorporating nonlinearities into the feedback model, Ringwood and Malpas determined that oscillatory behavior at low frequencies (~0.3Hz) can be maintained via nonlinear input-output relationships within the CNS over a wide range of gain changes that could occur at various points in the feedback loop. In other words, nonlinearities in the CNS afford greater stability to the oscillatory behavior of blood pressure.

Additional baroreceptor modeling efforts have further emphasized the nonlinear nature of the baroreceptor response to blood pressure changes (Henning & Khalil 1989; Ursino & Magosso 2000; Kawada et al. 1999). Interestingly, it has been shown in closed-loop simulations of baroreflex regulation of heart rate, baroreceptor, sympathetic, and parasympathetic nerve firing follows a hysteresis curve (time dependence of system outputs to present and past inputs). Moreover, the hysteresis loop associated with model conditions simulating healthy normotensive adults is wider than those associated with model conditions simulating hypertensive cases. This suggests some maladaptation (or lack of adaptation) in conditions associated with hypertension development (Henning & Khalil 1989).

A key physiological component to cardiovascular system that affects blood flow and pressure is the vasculature or venous system. Despite its complexity, many models have successfully utilized simple yet effective models that rely on concepts from electrical circuit design to characterize venous blood flow. A commonly used model that captures key hemodynamic properties is the Windkessel model (Westerhof et al. 2009). Originally used to describe aortic blood flow from the heart, it likens the heart and aortic flow to a closed hydraulic circuit that includes a water pump (i.e. heart) connected to a chamber. This modeling approach has been used effectively to incorporate hemodynamic effects on blood pressure (Degeest et al. 1965).

In addition, the respiratory system interacts with the cardiovascular system and affects heart rate variability. Although an examination of the cardiorespiratory system is beyond the scope of this work, modeling work has shown that respiratory sinus arrhythmia has beneficial effects, including reducing the work load of the heart while maintaining physiological levels of CO_2_ (Mastitskaya et al. 2012).

Finally, several previous efforts have integrated these neurogenic and physiological mechanisms into more comprehensive models. An example of such a model is a closed-loop hemodynamic model of baroreceptor reflex regulation of arterial blood pressure developed by Ursino (Ursino 1998). This model is of particular interest because this model includes many of the physiological and neural regulatory mechanisms involved in regulating arterial blood pressure. Multiple cardiovascular effector functions are included such as pulsatile heart rate, cardiac contractility, hemodynamics, and autonomic regulation of vascular system properties like vascular resistance. In addition, compartmentalized physiological structures are integrated into this model in order to provide a more accurate representation of the physiological components affecting hemodynamics and blood pressure. For example, Ursino incorporated a compartmentalized vascular system composed of components characterized by distinct capacitances; resistances; and unstressed volumes. This model has since been modified to study cardiovascular functions under various conditions, including exercise-induced stress (Magosso & Ursino 2002), and univentricular flow resulting from right heart-bypass operations as a treatment for congenital heart disease (Ursino & Magosso 2000).

**Extended Model Structure and Parameters**

The model described in this paper is an extension of a previous quantitative models originally developed by Ursino (1998), who used the model to examine the interaction between carotid baroregulation and the pulsating heart (Ursino 1998). Subsequent revisions to this model have been made by Ursino, et al. to explore various aspects of carotid baroregulation, respiration, and how this physiological control system responds to various stressors. A combination of various versions of this model (Ursino 1998; Ursino & Magosso 2000; Magosso & Ursino 2002; Magosso et al. 2002) are used in order to capture hemodynamic characteristics necessary to incorporate neuronal components driven by afferent input types. Because multiple aspects of previous models were integrated to develop the extended model described in this work, we felt it was important to provide a comprehensive description of the quantitative model in one place.

*We would like to emphasize that this text is based on previous descriptions from source material (Ursino 1998, Magosso & Ursino 2000, and Magosso et al. 2002).* *Interested readers are recommended to review these papers for detailed description of these models.*

*The model, created in the SIMULINK platform and associated Matlab code are available upon request.*

Conservation of mass and force balance equations used to characterize hemodynamics throughout the cardiovascular system are reproduced here. The following equations use the following variables to characterize hemodynamic behavior. Note that the subscript *j* represents the *j^th^* compartment.

1. S.1 Hemodynamic variables

| **Model parameters** | **Corresponding physiological parameter** |
| --- | --- |
| $P_{j}$ | Intravascular pressure |
| $V_{u,j}$ | Unstressed volume |
| $F_{j}$ | Blood flow |
| $C_{j}$ | Compliance |
| $L_{j}$ | Inertance |
| $R_{j}$ | Resistances |
| $F_{o,r}$ | Flow out of right ventricle |
| $F_{o,l}$ | Flow out of left ventricle |

**Vascular system**

Conservation of mass at pulmonary arteries *(pa)*

|  | $\frac{{dP}_{pa}}{dt}=\frac{1}{C_{pa}}\left( F_{o,r}-F_{pa} \right)$ | S.1 |
| --- | --- | --- |

Balance of forces at pulmonary arteries *(pa)*

|  | $\frac{{dF}_{pa}}{dt}=\frac{1}{L_{pa}}\left( P_{pa}-P_{pp}- R_{pa}*F_{pa} \right)$ | S.2 |
| --- | --- | --- |

The inertance represents the change in pressure required to cause a change in flow-rate of a fluid. Due to the large diameter of the arteries, inertances affect hemodynamic behavior more noticeably in these blood vessels than in the smaller diameter veins.

Conservation of mass at pulmonary peripheral circulation *(pp)*

|  | $\frac{{dP}_{pp}}{dt}=\frac{1}{C_{pa}}\left( F_{pa}-\frac{P_{pp}- P_{pv}}{R_{pp}} \right)$ | S.3 |
| --- | --- | --- |

Conservation of mass at pulmonary veins *(pv)*

|  | $\frac{{dP}_{pv}}{dt}=\frac{1}{C_{pv}}\left( \frac{P_{pp}- P_{pv}}{R_{pp}}-\frac{P_{pv}- P_{la}}{R_{pv}} \right)$ | S.4 |
| --- | --- | --- |

Conservation of mass at systemic arteries *(sa)*

|  | $\frac{{dP}_{sa}}{dt}=\frac{1}{C_{sa}}\left( F_{o,l}-F_{sa} \right)$ | S.5 |
| --- | --- | --- |

Force balance at systemic arteries

|  | $\frac{{dF}_{sa}}{dt}=\frac{1}{L_{sa}}\left( P_{sa}-P_{sp}- R_{sa}*F_{sa} \right)$ | S.6 |
| --- | --- | --- |

Conservation of mass at peripheral systemic circulation – splanchnic, extrasplanchnic, and lower body compartments *(sp, ep, mp)*

|  | $\frac{{dP}_{sp}}{dt}=\frac{1}{C_{sp}+C_{ep}+C_{mp}}*\left( F_{sa}-\frac{P_{sp}- P_{sv}}{R_{sp}}-\frac{P_{sp}- P_{ev}}{R_{ep}}-\frac{P_{sp}- P_{mv}}{R_{lb}} \right)$ | S.7 |
| --- | --- | --- |

Where

|  | $\left( R_{lb}= \frac{1}{R_{mp}}+ \frac{1}{R_{d}} \right)$ | S.7.1 |
| --- | --- | --- |

NOTE: To incorporate the effects of varying abdominal pressure $P_{abd}$ due to respiration, transmural pressure is calculated by subtracting $P_{abd}$ from $P_{sp}$. Equations for $P_{abd}$ are provided in later equations.

|  | $P_{sp-trans}= P_{sp}- P_{abd}$ | S.7.2 |
| --- | --- | --- |

This transmural pressure is subsequently used to determine downstream pressures.

Conservation of mass at extrasplanchnic venous circulation *(ev)*

|  | $\frac{{dP}_{ev}}{dt}=\frac{1}{C_{ev}}\left( \frac{P_{sp}- P_{ev}}{R_{ep}}-\frac{P_{ev}- P_{tv}}{R_{ev}}-\frac{{dV}_{u,ev}}{dt} \right)$ | S.8 |
| --- | --- | --- |

Conservation of mass at skeletal muscle, part of the lower body *(mv)*

|  | $\frac{{dP}_{mv}}{dt}=\frac{1}{C_{mv}}\left( \frac{P_{sp}- P_{mv}}{R_{lb}}-\frac{P_{mv}- P_{tv}}{R_{mv}}-\frac{{dV}_{u,mv}}{dt} \right)$ | S.9 |
| --- | --- | --- |

Conservation of mass at thoracic vein *(tv)*

|  | $\frac{{dP}_{tv}}{dt}=\frac{1}{C_{tv}}\left( \frac{P_{mv}- P_{tv}}{R_{mv}}+\frac{P_{ev}- P_{tv}}{R_{ev}}+\frac{P_{sv}- P_{tv}}{R_{ev}}-\frac{P_{tv}- P_{ra}}{R_{tv}} \right)$ | S.10 |
| --- | --- | --- |

NOTE: To incorporate the effects of varying thoracic pressure $P_{thor}$ due to respiration, transmural pressure is calculated by subtracting $P_{thor}$ from $P_{tv}$. Equations for $P_{thor}$ are provided in later equations.

|  | $P_{tv-trans}= P_{tv}- P_{thor}$ | S.10.1 |
| --- | --- | --- |

Conservation of mass determines splanchnic venous circulation, which assumes total blood volume *(*$V_{t}$*)* is known.

|  | $P_{sv}=\frac{1}{C_{sv}}\left( V_{t}-C_{sa}*P_{sa}-\left( C_{sp}+C_{ep}+C_{mp} \right)*P_{sp}-C_{ev}*P_{ev}-C_{mv}*P_{mv}-{C_{tv}*P_{tv}-C}_{ra}*P_{ra}-V_{ra}-C_{pa}*P_{pa}-C_{pp}*P_{pp}-C_{pv}*P_{pv}-C_{la}*P_{la}-V_{lv}-V_{u} \right)$ | S.11 |
| --- | --- | --- |

Here, $V_{rv}$ and $V_{lv}$ are the volumes of the right and left ventricles. $V_{u}$ is the total unstressed volume, defined by:

**Pulsatile (left) heart**

Conservation of mass at left atrium (la)

|  | $\frac{{dP}_{la}}{dt}=\frac{1}{C_{la}}\left( \frac{P_{pv}- P_{la}}{R_{pv}}-F_{i,l} \right)$ | S.12 |
| --- | --- | --- |

$F_{i,l}$refers to the flow into the left ventricle, determined by the following mass balance:

|  | $F_{i,l}=\left\{ \begin{aligned} 0, &P_{la}<P_{lv} \\ \frac{P_{la}- P_{lv}}{R_{la}}, &P_{la}\geq P_{lv} \end{aligned} \right.$ | S.13 |
| --- | --- | --- |

Ventricular volume is calculated using the following equation:

|  | $\frac{{dV}_{lv}}{dt}=F_{i,l}-F_{o,l}$ | S.14 |
| --- | --- | --- |

And flow out of the left ventricle *(*$F_{o,l}$*)* is determined by:

|  | $F_{o,l}=\left\{ \begin{aligned} 0, &P_{max,lv}<P_{sa} \\ \frac{P_{max,lv}- P_{sa}}{R_{lv}}, &P_{max,lv}\geq P_{sa} \end{aligned} \right.$ | S.15 |
| --- | --- | --- |

$P_{max,lv}$ represents the isometric pressure of the left ventricle. This value is used to determine ventricular pressure over the course of the cardiac cycle. $R_{lv}$ represents the viscous resistance of the left ventricle and is assumed to be proportional to $P_{max,lv}$ where:

|  | $R_{lv}=k_{R,lv}*P_{max,lv}$ | S.16 |
| --- | --- | --- |

$k_{R,lv}$is a constant parameter.

Instantaneous pressure in the left ventricle represents the difference between the isometric pressure ($P_{max,lv}$) and viscous losses, therefore:

|  | $P_{lv}=P_{max,lv}-R_{lv}*F_{o,l}$ | S.17 |
| --- | --- | --- |

Isometric pressure is time-dependent and varies throughout the cardiac cycle. The base model by Ursino assumes that isometric pressure/volume can be characterized by an exponential function during diastole, when the ventricle is relaxed, and by a linear function at the end of systole, when the ventricle is contracted maximally. Thus isometric pressure transitions between an exponential and linear function over the course of the cardiac cycle.

|  | $P_{max,lv}\left( t \right)-\varphi\left( t \right)*E_{max,lv}*\left( V_{lv}-V_{u,lv} \right)+\left[ 1-\varphi\left( t \right) \right]*P_{0,lv}*(\exp\left( k_{E,lv}*V_{lv} \right)-1$ | S.18 |
| --- | --- | --- |

Where $0 \leq\varphi\left( t \right)\leq1$

$E_{max,lv}$ is the ventricular elastance at the maximal contraction of the ventricle. $V_{u,lv}$ is the corresponding unstressed volume of the ventricle and is the x-axis intercept of the end-systolic pressure/volume function. $P_{0,lv}$ and $k_{E,lv}$ are constant parameters that describe the monoexponential pressure/volume function at diastole.

The term $\varphi\left( t \right)$ represents the “activation function” of the ventricle. When $\varphi\left( t \right)=1$, the ventricle is at maximum contraction, when $\varphi\left( t \right)=0$, it is at complete relaxation. This activation function is defined as:

|  | $\varphi\left( t \right)=\left\{ \begin{aligned} \sin^{2} \left[ \frac{\pi*T\left( t \right)}{T_{sys}\left( t \right)}*u \right], 0\leq u\leq\frac{T_{sys}}{T} \\ 0, & \frac{T_{sys}}{T}\leq u\leq1 \end{aligned} \right.$ | S.19 |
| --- | --- | --- |

$T$ represents the heart period (i.e. inverse of heart rate). $T_{sys}$ is the duration of systole and u is a dimensionless variable ranging between 0 and 1 and represents the fraction of the cardiac cycle. A value of *u* = 0 corresponds to the beginning of systole. This variable has been modeled as an integral pulse frequency modulation function (Bailón et al. 2011).

|  | $u\left( t \right)=frac\left[ \int_{t_{0}}^{t} \frac{1}{T\left( t \right)}d\tau+u\left( t_{0} \right) \right]$ | S.20 |
| --- | --- | --- |

The fractional part of this equation, frac[ ], indicates that the variable $u\left( t \right)$ is reset to zero as soon as the value reaches a value of 1.

The duration of systole is determined by the following equation:

|  | $T_{sys}=T_{sys,0}-k_{sys}*\frac{1}{T}$ | S.21 |
| --- | --- | --- |

Where $k_{sys}$ and $T_{sys,0}$ are constant parameters. A separate set of equations similar to equations S.13-S.22 are used to describe the right heart as well.

**Pulsatile (right) heart**

Conservation of mass at right atrium (ra)

|  | $\frac{{dP}_{ra}}{dt}=\frac{1}{C_{ra}}\left( \frac{P_{tv}- P_{ra}}{R_{tv}}-F_{i,l} \right)$ | S.22 |
| --- | --- | --- |

Flow into right ventricle:

|  | $F_{i,r}=\left\{ \begin{aligned} 0, &P_{ra}<P_{rv} \\ \frac{P_{ra}- P_{rv}}{R_{ra}}, &P_{ra}\geq P_{rv} \end{aligned} \right.$ | S.23 |
| --- | --- | --- |

Volume of right ventricle:

|  | $\frac{{dV}_{rv}}{dt}=F_{i,r}-F_{o,r}$ | S.24 |
| --- | --- | --- |

Flow out of right ventricle:

|  | $F_{o,r}=\left\{ \begin{aligned} 0, &P_{max,rv}<P_{pa} \\ \frac{P_{max,rv}- P_{pa}}{R_{rv}}, &P_{max,rv}\geq P_{pa} \end{aligned} \right.$ | S.25 |
| --- | --- | --- |

Viscous resistance of right ventricle:

|  | $R_{rv}=k_{R,rv}*P_{max,rv}$ | S.26 |
| --- | --- | --- |

Instantaneous pressure in right ventricle:

|  | $P_{rv}=P_{max,rv}-R_{rv}*F_{o,r}$ | S.27 |
| --- | --- | --- |

Isometric pressure in right ventricle:

|  | $P_{max,rv}\left( t \right)-\varphi\left( t \right)*E_{max,rv}*\left( V_{rv}-V_{u,rv} \right)+\left[ 1-\varphi\left( t \right) \right]*P_{0,rv}*(\exp\left( k_{E,rv}*V_{rv} \right)-1$ | S.28 |
| --- | --- | --- |

Where $\varphi\left( t \right)$ is determined from equations S.20-S.22.

**Afferent input types**

*Baroreceptors* are modeled using a linear derivative first-order dynamic function and a sigmoidal static characteristic function in series, described by the following equations:

|  | $\tau_{p}\frac{d\acute{P}}{dt}=P_{br}+\tau_{z}*\frac{dP_{br}}{dt}-\acute{P}$ | S.29 |
| --- | --- | --- |
|  | $f_{br}=\left[ f_{min}+f_{max}*exp\left( \frac{\acute{P}-P_{n}}{k_{a}} \right) \right]/\left[ 1+exp\left( \frac{\acute{P}-P_{n}}{k_{a}} \right) \right]$ | S.30 |

Here, $\tau_{p}$and $\tau_{z}$ are time constants for the real pole and real zero in the linear dynamic block. $P_{br}$ is the arterial pressure measured by the baroreceptors. $\acute{P}$ is the output variable of the dynamic block (with dimensions of pressure). $f_{br}$ is the frequency of spikes in the afferent fibers. $f_{min}$and $f_{max}$ are the lower and upper saturation limits of the frequency discharge of the baroreceptors. $P_{n}$ is the intrasinus pressure at the central point of the sigmoidal curve and $k_{a}$is a constant parameter (with dimensions of pressure).

*Cardiopulmonary receptors* are modeled using a first-order low-pass filter in series with the same sigmoidal static characteristic function type used to model the baroreceptors. Because cardiopulmonary receptors depend on transmural pressure at the pulmonary veins, this pressure difference is used as an input to first-order low-pass filter:

|  | $\tau_{cp}\frac{dP_{l}}{dt}={-P}_{l}+\left( P_{pv}-P_{thor} \right)$ | S.31 |
| --- | --- | --- |
|  | $f_{cp}=\frac{f_{max,l}}{1+exp\left( \frac{P_{tn}-P_{l}}{k_{l}} \right)}$ | S.32 |

Here $P_{pv}-P_{thor}$ is the transmural pressure at the pulmonary vein or input value to the low-pass filter function. $P_{l}$ is the output variable of the low-pass filter. $f_{cp}$ is the spike frequency of the afferent fibers from the cardiopulmonary receptors and $f_{max,l}$ is the upper saturation limit of the frequency discharge of these receptors, the lower limit being zero. $P_{tn}$ represents the pulmonary venous pressure at the central point of the sigmoid curve. $k_{l}$ is another constant that determines the slope of the sigmoid curve, or sensitivity of the cardiopulmonary receptors.

*Lung stretch receptors* are modeled using a first-order low-pass filter:

|  | $\frac{df_{lr}}{dt}=\tau_{lung}*\left( -f_{lr}+G_{al}*V_{lung} \right)$ | S.33 |
| --- | --- | --- |

Here $f_{lr}$ is the firing discharge rate of the slowly adapting lung stretch receptors (SARs). $\tau_{lung}$ is the time constant of the receptor response to lung inflation. $G_{al}$ is a constant gain factor and $V_{lung}$is the lung volume.

**Integration of afferent input types in brainstem**

Afferent input types are combine in a linear manner prior to being received by the respective brainstem nuclei such as the nucleus ambiguus (NA) and dorsal motor nucleus of the vagus (DMV), included in the model. As a reminder, each brain nucleus is represented as a transfer function (main text, eqn 1):

|  | $f_{out,j}=\frac{f_{min,j}+ f_{max},j*exp\left( \frac{f_{input,j}-f_{midpt,j}}{k} \right)}{1+\exp\left( \frac{f_{input,j}-f_{midpt,j}}{k,j} \right)}$ | eqn. 1 |
| --- | --- | --- |

As described in the “*NTS, NA, and the DMV Function and Role in Parasympathetic (Vagal) Outflow*” in the main text, the NA and DMV receive a linear combination of firing frequency signals generated by the NTS neuronal subtype:

|  | $f_{input,NA}=K_{br,NA}f_{br}+ K_{cp,NA}f_{cp}+ K_{lr,NA}f_{lr}$ | S.34 |
| --- | --- | --- |
|  | $f_{input,DMV}=K_{br,DMV}f_{br}+ K_{cp,DMV}f_{cp}+ K_{lr,DMV}f_{lr}$ | S.35 |

$K_{xx,YY}$ represents a gain factor, or weight, of the firing frequency signal from the respective NTS neuronal subtype *(br, cp, lr*) to the respective brainstem nuclei (*NA, DMV*). Note that the $K_{br,DMV}$ is set to 0 to remove any influence of baroreceptors have on the DMV. Concomitantly, a $K_{lr,DMV}$ is set to 1 in order to facilitate an effect of lung tidal volume (mediated by lung stretch receptors in the NTS) on heart contractility, which is predominantly regulated by the DMV. This connection between the lung stretch receptors and DMV is thus an imposition placed in the model to maintain the overall relationship observed experimentally (Greenwood et al. 1980; Hainsworth 1974).

**Efferent sympathetic outflow**

Sympathetic efferent outflow is modeled to be dependent on the afferent input signals sent by the baroreceptor, cardiopulmonary, and lung stretch receptors. Moreover, the combined effects of these input signals affect sympathetic efferent outflow to different effector functions differently. Therefore a series of calculations are included to determine *i)* the afferent firing frequency input that is then used to determine *ii)* the distinct sympathetic efferent outflow signals to the respective effector functions associated with the heart (*h*), peripheral circulation (*p*), and unstressed volumes (*v*)

|  | $f_{as, h}={G_{ab,h}*f}_{br}-{G_{alh}*f}_{lr}+{G_{ac,h}*f}_{cp}$ | S.36 |
| --- | --- | --- |
|  | $f_{as,p}={G_{ab,p}*f}_{br}+{G_{al,p}*f}_{lr}+{G_{ac,p}*f}_{cp}$ | S.37 |
|  | $f_{as, v}={G_{ab,v}*f}_{br}+{G_{al,v}*f}_{lr}+{G_{ac,v}*f}_{cp}$ | S.38 |

$G_{ab,j}$, where j is a general index for the heart, peripheral circulation, or unstressed volume, represents a constant gain factor indicating how much influence each afferent input has on determining sympathetic efferent outflow. A factor of -1 is used for $G_{al,h}$ as this provided the best fits for the model. The resulting $f_{as,j}$ values are then used to determine sympathetic efferent outflow to the respective effector functions using a negative monotonic function to relate afferent activity to efferent neural pathways,

|  | $f_{es,h}=f_{es,\infty}+\left( f_{es,0}-f_{es,\infty} \right)*exp\left( {-k}_{es}*f_{as,h} \right)$ | S.39 |
| --- | --- | --- |
|  | $f_{es,p}=f_{es,\infty}+\left( f_{es,0}-f_{es,\infty} \right)*exp\left( {-k}_{es}*f_{as,p} \right)$ | S.40 |
|  | $f_{es,v}=f_{es,\infty}+\left( f_{es,0}-f_{es,\infty} \right)*exp\left( {-k}_{es}*f_{as,v} \right)$ | S.41 |

**Parasympathetic (vagal) efferent output** is described in detail in the main text.

**Effector function regulation**

Physiological parameters affected by sympathetic and parasympathetic outflow include resistances, unstressed volumes, and cardiac elastances. Sympathetic outflow regulates resistances and unstressed volumes via a monotonic logarithmic static function, a low-pass first-order dynamics, and a time delay specific to each effector function.

|  | $\sigma_{\theta}\left( t \right)=\left\{ \begin{aligned} G_{\theta}*ln\left[ f_{es,j}\left( t-D_{\theta} \right)-f_{es,min}+1 \right], &f_{es,j}\geq f_{es,min} \\ 0 , f_{es,j}<f_{es,min} \end{aligned} \right.$ | S.42 |
| --- | --- | --- |
|  | $\frac{d\Delta\theta}{dt}\left( t \right)=\frac{1}{\tau_{\theta}}*\left( -\Delta\theta\left( t \right)+\sigma_{\theta}\left( t \right) \right)$ | S.43 |
|  | $\theta\left( t \right)=\Delta\theta\left( t \right)+\theta_{0}$ | S.44 |

Where θ represents generic controlled parameters (i.e. resistance or unstressed volume). $\tau_{\theta}$ and $D_{\theta}$ are the time constants and time delays associated with sympathetic regulatory mechanisms on these effector functions. $G_{\theta}$ is a constant gain factor for the various effector functions and *θ_0_* represents constant values for respective effector functions. Note that $f_{es,j}$ is used to represent the different sympathetic tones specific to a particular effector function.

**Heart period**

In the original model developed by Ursino (1998), heart period, as opposed to heart rate, is modeled. By modeling heart period, Ursino was able to reproduce the nonlinear effect that sympathetic and vagal tone have on heart rate. Thus a linear interaction between sympathetic and parasympathetic effect on heart period is used. Heart period (not heart rate) has been shown to be linearly dependent on vagal drive, while the same monotonic logarithmic static function and low-pass first-order dynamics characterize sympathetic effects on heart period.

|  | $\sigma_{T,s}\left( t \right)=\left\{ \begin{aligned} G_{T,s}*ln\left[ f_{es,h}\left( t-D_{\theta} \right)-f_{es,min}+1 \right], &f_{es,h}\geq f_{es,min} \\ 0 , f_{es,h}<f_{es,min} \end{aligned} \right.$ | S.45 |
| --- | --- | --- |
|  | $\frac{d\Delta T_{s}}{dt}\left( t \right)=\frac{1}{\tau_{T,s}}*\left( -\Delta T_{s}\left( t \right)+\sigma_{T,s}\left( t \right) \right)$ | S.46 |
|  | $\sigma_{T,v}=G_{T,v}*f_{ev,h}\left( t-D_{T,v} \right)$ | S.47 |
|  | $\frac{d\Delta T_{v}}{dt}\left( t \right)=\frac{1}{\tau_{T,v}}*\left( -\Delta T_{v}\left( t \right)+\sigma_{T,v}\left( t \right) \right)$ | S.48 |
|  | $T=\Delta T_{s}+\Delta T_{v}+T_{0}$ | S.49 |

**Ventricular Contractility**

Because ventricular contractility is dependent on the balance of sympathetic and parasympathetic drive, similar to heart period, a similar approach is used to determine contractility. Thus a linear interaction between sympathetic and parasympathetic effects on the inverse of $E_{max,lv}$ is used

|  | $\sigma_{E,s}\left( t \right)=\left\{ \begin{aligned} {-G}_{s,Emax}*ln\left[ f_{es,h}\left( t-D_{E,s} \right)-f_{es,min}+1 \right], &f_{es,h}\geq f_{es,min} \\ 0 , f_{es,h}<f_{es,min} \end{aligned} \right.$ | S.50 |
| --- | --- | --- |
|  | $\frac{d\Delta\left( \frac{1}{E_{max,lv}} \right)_{s}}{dt}\left( t \right)=\frac{1}{\tau_{E,s}}*\left( -\Delta\left( \frac{1}{E_{max,lv}} \right)_{s}\left( t \right)+\sigma_{E,s}\left( t \right) \right)$ | S.51 |
|  | $\sigma_{E,v}=G_{E,v}*f_{ev,h}\left( t-D_{E,v} \right)$ | S.52 |
|  | $\frac{d\Delta\left( \frac{1}{E_{max,lv}} \right)_{v}}{dt}\left( t \right)=\frac{1}{\tau_{E,v}}*\left( -\Delta\left( \frac{1}{E_{max,lv}} \right)_{v}\left( t \right)+\sigma_{E,v}\left( t \right) \right)$ | S.53 |
|  | $\left( \frac{1}{E_{max,lv}^{'}} \right)=\Delta\left( \frac{1}{E_{max,lv}} \right)_{s}+\Delta\left( \frac{1}{E_{max,lv}} \right)_{v}$ | S.54 |
|  | $E_{max,lv}=E_{max,lv}^{'}+E_{max,lv,0}$ | S.55 |

Here, $\tau_{E,s}$, and $\tau_{E,v}$, represent the time constants associated with the sympathetic and parasympathetic regulatory mechanisms on contractility. $D_{E,s}$ and $D_{E,v}$ correspond to the time delays associated the sympathetic and parasympathetic mechanisms. Once $\frac{1}{E_{max,lv}^{'}}$is determined, $E_{max,lv}$ can easily be calculated using equation S.55. $E_{max,lv,0}$ represents a constant, baseline elasticity value determined from experimental data collected from a dog whose stellate ganglion and vagal nerve fibers were denervated, effectively removing any autonomic influence (Suga et al. 1976).

Equations similar to S.50-S.55 are used to determine $\frac{1}{E_{max,rv}}$ (right ventricle). However, all gains were adjusted by a factor of (1/0.59), based on the ratio value used to relate contractility between the right and left ventricle used by Ursino originally.

**Lung volume, thoracic, and abdominal pressures**

A linear relationship between lung volume and thoracic pressure is used to model lung volume measured by the lung stretch receptors:

|  | $V_{lung}=V_{lung,0}-0.1*P_{thor}$ | S.56 |
| --- | --- | --- |

Thoracic pressure varies with time due to the effects of the respiratory cycle, which is modeled independently from any autonomic regulation. Parameters were chosen based on experimental work by Moreno, et al. (1969). Thoracic pressure varies linearly during respiration between a minimum of −9 mmHg and a maximum of −4 mmHg, which represents steady-state thoracic pressure during the respiratory pause, as modeled by the following equations:

|  | $P_{thor}=\left\{ \begin{aligned} P_{thor, max}-\left( P_{thor, max}-P_{thor, min} \right)*\frac{T_{resp}}{T_{insp}}*s 0 \leq s\leq\frac{T_{insp}}{T_{resp}}*s \\ P_{thor, max}-\frac{\left( P_{thor, max}-P_{thor, min} \right)}{T_{exp}}*\left( T_{insp}-T_{exp}-s*T_{resp} \right), \frac{T_{insp}}{T_{resp}} \leq s\leq\frac{T_{insp}+T_{exp}}{T_{resp}} \\ P_{thor, max} \frac{T_{insp}+T_{exp}}{T_{resp}}\leq s\leq1 \end{aligned} \right.$ | S.57 |
| --- | --- | --- |

Where $T_{resp}$ is the period of respiration. $T_{insp}$ is the inspiration time,$T_{exp}$ is the expiration time. $P_{thor, min}$is the value of the intrathoracic pressure at the end of inspiration while $P_{thor, max}$ is the max value of intrathoracic pressure at the end of expiration and throughout the respiratory pause, which takes place in between each respiration period. The parameter $s$ is dimensionless, varying between 0 and 1, and represents the fraction of the respiratory cycle. A value of 0 represents the beginning of the respiration cycle. Similar to other parameters representing a fraction of cycle completion, an expression for $s(t)$has been developed by Magosso et al. (2001) by introducing yet another state variable $\varepsilon(t)$, which is determined by the following equation

| $\frac{d\varepsilon}{dt}=\frac{1}{T_{resp}} with s\left( t \right) =frac(\varepsilon)$ | S.58 |
| --- | --- |

Abdominal pressure is modeled accordingly:

|  | $P_{abd}=\left\{ \begin{aligned} \begin{aligned} -2.5*s&*\frac{T_{resp}}{{T_{insp}}/2}-4 0<s<\frac{{T_{insp}}/2}{T_{resp}} \\ -2.5 \frac{{T_{insp}}/2}{T_{resp}}<s<\frac{T_{insp}}{T_{resp}} \end{aligned} \\ -2.5*\frac{T_{insp}+T_{exp}-s*T_{resp}}{T_{exp}} \frac{T_{insp}}{T_{resp}}<s<\frac{T_{insp}+T_{exp}}{T_{resp}} \\ -5*\frac{T_{insp}+T_{exp}-s*T_{resp}}{T_{exp}}-4 \frac{T_{insp}+T_{exp}}{T_{resp}}<s<1 \end{aligned} \right.$ | S.59 |
| --- | --- | --- |

Where $T_{resp}$ represents the respiratory period, $T_{insp}$ represents the duration of inspiration and $T_{exp}$ represents the duration of expiration. $s$ is a dimensionless variable, similar to the variable $u(t)$, used to represent the fraction of the cardiac cycle that has completed. Here, $s$ is calculated by solving for an additional state variable, $\varepsilon$

|  | $\frac{d\varepsilon}{dt}=\frac{1}{T_{resp}}$ | S.60 |
| --- | --- | --- |
|  | $s\left( t \right)=frac\left( \varepsilon\right)$ | S.61 |

Where the fractional portion $frac\left( \varepsilon\right)$ resets the variable $s\left( t \right)$ to zero once it reaches a value of 1.

**Simulation of Exercise Conditions**

When simulating exercise conditions, several changes occur relative to conditions representing baseline physiology. Firstly, intramuscular pressure ($P_{im}$), the extravascular pressure outside of the active muscle veins, exhibits a periodic pattern (Mag0sso et al. 2002). Following the work of Magosso et al. (2002), we use the same equations to simulate $P_{im}$:

|  | $P_{im}=A*\Psi(t)$ | S.62 |
| --- | --- | --- |
|  | $\Psi(t)=\left\{ \begin{aligned} sin\left( \pi*\frac{T_{im}}{T_{c}}*\alpha\right), 0\leq\alpha\leq\frac{T_{im}}{T_{c}} \\ 0, \frac{T_{im}}{T_{c}} \leq\alpha\leq1 \end{aligned} \right.$ | S.63 |

Here, $A$ represents the peak value of intramuscular pressure, which is the value of $P_{im}$ at the instant of maximum contraction. $\Psi(t)$ represents the activation function of skeletal muscle fibers and varies between values of 0 (complete muscle relaxation) and 1 (maximum muscle contraction). $T_{im}$ represents the duration of muscle contraction-relaxation cycle and $T_{c}$ is the overall duration of contraction. The final constant in this set of equations is $\alpha$, which represents the fraction of muscular contraction-relaxation cycle and varies between 0 (beginning of contraction) and 1 (end of contraction). Similar to heart rate and other cardiovascular outputs that involve model parameters that follow a period pattern, Magosso et al. introduce another state parameter $\zeta(t)$, to determine $\alpha(t)$:

| $\frac{d\zeta}{dt}=\frac{1}{T_{im}} with \alpha\left( t \right) =frac(\zeta)$ | S.64 |
| --- | --- |

The fractional part of this equation, frac[ ], indicates that the variable $\alpha\left( t \right)$is reset to zero as soon as its value reaches 1.

Secondly, under exercise conditions, blood flow will be divided differently as an increase in blood flow to the skeletal muscles of the leg, as cardiopulmonary performance is tested via exercise bike. Following Magosso et al. (2002), blood flow to the extrasplanchnic compartment is divided into parallel flows, which include a reduced extrasplanchnic compartment and an active muscle compartment, which in this case represent the skeletal muscles of the leg because cardiopulmonary performance is often measured via bicycling activity on a ergometer. We use the same division of cardiac output (CO), where ~13% of CO is diverted to the skeletal muscles of the leg and blood flow to the rest of the extrasplanchnic vascular beds is ~ 57% of CO (Magosso et al. 2002). Also, as strong muscular contractions may cause veins to collapse, due to the “muscle pump”, the relationship between transmural pressure and blood volume in the active muscle veins has been modeled as a collapsible tube (Magosso et al. 2002):

Muscle “pump”

|  | $P_{mv}- P_{im}=\left\{ \begin{aligned} C_{mv}*\left( V_{mv}-V_{u,mv} \right) V_{mv}>V_{u,mv} \\ P_{0}*\left[ 1-\left( \frac{V_{mv}}{V_{u,mv}} \right)^{\frac{-3}{2}} \right] V_{mv}<V_{u,mv} \end{aligned} \right.$ | S.65 |
| --- | --- | --- |

Here, $P_{mv}$ represents the pressure inside active muscle veins and $P_{im}$is the extravascular pressure of the active muscle veins (i.e. transmural pressure). Under resting conditions, $P_{im}$is zero, under active conditions, $P_{im}$changes periodically. Additional parameters include $V_{mv}$, the total blood volume (sum of stressed and unstressed volumes) in the active muscle veins. $V_{u,mv}$ is the unstressed volume of the active muscle veins, $C_{mv}$ is the venous compliance in the active muscle compartment and $P_{0}$ is a constant parameter. Per Magosso et al. (2002), $P_{0}= V_{u,mv}/\left( C_{mv}*10 \right)$, which equates to 3.9.

Blood flow through the active muscle compartment is modeled as a diode arranged in series with hydrodynamic resistance due to the active muscle veins. The following equations to model this arrangement are as follows:

|  | $F_{o,m}=\left\{ \begin{aligned} 0, &P_{mv}\leq P_{tv} \\ \frac{P_{mv}- P_{tv}}{R_{mv}}, &P_{mv}>P_{tv} \end{aligned} \right.$ | S.66 |
| --- | --- | --- |

Where $P_{tv}$ is the pressure inside the thoracic veins.

**Supplemental References**

Bailón, R. et al., 2011. The integral pulse frequency modulation model with time-varying threshold: Application to heart rate variability analysis during exercise stress testing. *IEEE Transactions on Biomedical Engineering*, 58(3 PART 1), pp.642–652.

Ben-Tal, A., Shamailov, S.S. & Paton, J.F.R., 2014. Central regulation of heart rate and the appearance of respiratory sinus arrhythmia: New insights from mathematical modeling. *Mathematical Biosciences*, 255(1), pp.71–82. Available at: http://dx.doi.org/10.1016/j.mbs.2014.06.015.

Burgess, D.E. et al., 1997. First-order differential-delay equation for the baroreflex predicts the 0.4-Hz blood pressure rhythm in rats. *Am J Physiol*, 273(6 Pt 2), pp.R1878-84. Available at: http://www.ncbi.nlm.nih.gov/pubmed/9435640.

Degeest, H. et al., 1965. Depression of Ventricular Contractility By Stimulation of the Vagus Nerves. *Circulation research*, 17(September 1965), pp.222–235.

Greenwood, P.V. et al., 1980. Reflex Inotropic Responses of the Heart from Lung Inflation in Anaesthetized Dogs. *European journal of Physiology*, 205, pp.199–205.

Hainsworth, R., 1974. Circulatory inflation responses in anesthetized from lung dogs. *American Journal of Physiology*, 226(2).

Henning, R.J. & Khalil, I., 1989. A u t o n o m i c nervous stimulation affects left ventricular relaxation more than left ventricular contraction. , 28, pp.15–25.

Kawada, T. et al., 1999. Simultaneous identification of static and dynamic vagosympathetic interactions in regulating heart rate. *The American journal of physiology*, 276, pp.R782–R789.

Magosso, E., Cavalcanti, S. & Ursino, M., 2002. Theoretical analysis of rest and exercise hemodynamics in patients with total cavopulmonary connection. *American journal of physiology. Heart and circulatory physiology*, 282(3), pp.H1018-34. Available at: http://www.ncbi.nlm.nih.gov/pubmed/11834500.

Magosso, E. & Ursino, M., 2002. Cardiovascular response to dynamic aerobic exercise: A methematical model. *Medical and Biological Engineering and Computing*, 40(6), pp.660–674. Available at: http://link.springer.com/article/10.1007/BF02345305%5Cnhttp://link.springer.com/article/10.1007%2FBF02345305?LI=true#page-1%5Cnhttp://link.springer.com/content/pdf/10.1007%2FBF02345305.

Mastitskaya, S. et al., 2012. Cardioprotection evoked by remote ischaemic preconditioning is critically dependent on the activity of vagal pre-ganglionic neurones. *Cardiovascular research*, 95(4), pp.487–94. Available at: http://cardiovascres.oxfordjournals.org/content/95/4/487.long [Accessed May 12, 2014].

Moreno, A.H., Katz, A.I. & Gold, L.D., 1969. An integrated approach to the study of the venous system with steps toward a detailed model of the dynamics of venous return to the right heart. *IEEE transactions on bio-medical engineering*, 16(4), pp.308–324.

Ringwood, J. V & Malpas, S.C., 2001. Slow oscillations in blood pressure via a nonlinear feedback model. *American journal of physiology. Regulatory, integrative and comparative physiology*, 280(4), pp.R1105–R1115.

Suga, H., Sagawa, K. & Kostiuk, D.P., 1976. Controls of ventricular contractility assessed by pressure-volume ratio, Emax. *Cardiovascular Research*, 10(5), pp.582–592.

Ursino, M., 1998. Interaction between carotid baroregulation and the pulsating heart: a mathematical model. *The American journal of physiology*, 275(5 Pt 2), pp.H1733–H1747.

Ursino, M. & Magosso, E., 2000. Acute cardiovascular response to isocapnic hypoxia. I. A mathematical model. *American journal of physiology. Heart and circulatory physiology*, 279(1), pp.H149–H165.

Westerhof, N., Lankhaar, J.W. & Westerhof, B.E., 2009. The arterial windkessel. *Medical and Biological Engineering and Computing*, 47(2), pp.131–141.
