# Supplementary material for: Investigating the Effects of Brainstem Neuronal Adaptation on Cardiovascular Homeostasis
Source: Front Neurosci. 2020 May 20;14:470. doi: 10.3389/fnins.2020.00470 (PMC7251082; doi:10.3389/fnins.2020.00470)
Supplement: TABLE S2 — Hemodynamic parameter values (vascular system). [file Table_2.docx]

Investigating the Effects of Brainstem Neuronal Adaptation on Cardiovascular Homeostasis

Supplemental Tables – Model Parameters

Extended model parameter values for basal (healthy) state

1. S.2 Hemodynamic parameter values (vascular system)

| **Parameter** | **Value** | **Reference** |
| --- | --- | --- |
| Compliances (mL/mmHg) | | |
| $C_{sa}$ | 0.28 | [1] |
| $C_{sp}$ | 2.05 | [1] |
| $C_{ep}$ | 1.36 | [2] |
| $C_{mp}$ | 0.31 | [2] |
| $C_{sv}$ | 43.11 | [2] |
| $C_{ev}$ | 28.40 | [2] |
| $C_{mv}$ | 6.60 | [2] |
| $C_{tv}$ | 33 | [2] |
| $C_{pa}$ | 0.76 | [1] |
| $C_{pp}$ | 5.80 | [1] |
| $C_{pv}$ | 25.37 | [1] |
| Unstressed Volumes (mL) | | |
| $V_{u,sa}$ | 0 | [1] |
| $V_{u,sp}$ | 274.40 | [1] |
| $V_{u,ep}$ | 274.1 | [2] |
| $V_{u,mp}$ | 62.50 | [2] |
| $V_{u,sv}$ | 1121 | [1] |
| $V_{u,ev}$ | 1120 | [2] |
| $V_{u,mv}$ | 255 | [2] |
| $V_{u.tv}$ | 0 | [3] |
| $V_{u.pa}$ | 0 | [1] |
| $V_{u,pp}$ | 123 | [1] |
| $V_{u,pv}$ | 120 | [1] |
| Hydraulic Resistances (mmHg*s*mL^-1^) | | |
| $R_{sa}$ | 0.06 | [1] |
| $R_{sp}$ | 3.307 | [1] |
| $R_{ep}$ | 1.725 | [2] |
| $R_{mp}$ | 4.130 | [2] |
| $R_{sv}$ | 0.038 | [1] |
| $R_{ev}$ | 0.0197 | [2] |
| $R_{mv}$ | 0.0848 | [2] |
| $R_{tv}$ | 0.0054 | [2] |
| $R_{pa}$ | 0.0230 | [1] |
| $R_{pp}$ | 0.0894 | [1] |
| $R_{pv}$ | 0.0056 | [1] |
| Inertance (mmHg*ml*s^-2^) | | |
| $L_{sa}$ | 2.2e-4 | [1] |
| $L_{pa}$ | 1.8e-4 | [1] |

1. S.3 Hemodynamic parameters (left heart)

| **Parameter** | **Value** | **Units** | **Reference** |
| --- | --- | --- | --- |
| $C_{la}$ | 19.23 | mL/mmHg | [1] |
| $V_{u,la}$ | 25 | mL | [1] |
| $R_{la}$ | 2.5e-3 | mmHg*s*mL^-1^ | [1] |
| $P_{0,lv}$ | 1.5 | mmHg | [1] |
| $k_{E,lv}$ | 0.014 | mL^-1^ | [1] |
| $V_{u,lv}$ | 16.77 | mL | [1] |
| $E_{max,lv,0}$ | 1.283 | mmHg/mL | estimated |
| $k_{R,lv}$ | 3.75e-4 | s/mL | [1] |

1. S.4 Activation function parameters

| **Parameter** | **Value** | **Units** | **Reference** |
| --- | --- | --- | --- |
| $k_{sys}$ | 0.075 | sec^2^ | [1] |
| $T_{sys,0}$ | 0.40 | sec | [1] |

1. S.5 Hemodynamic parameters (left heart)

| **Parameter** | **Value** | **Units** | **Reference** |
| --- | --- | --- | --- |
| $C_{ra}$ | 31.25 | mL/mmHg | [1] |
| $V_{u,ra}$ | 25 | mL | [1] |
| $R_{ra}$ | 2.5e-3 | mmHg*s*mL^-1^ | [1] |
| $P_{0,rv}$ | 1.5 | mmHg | [1] |
| $k_{E,rv}$ | 0.0110 | mL^-1^ | [1] |
| $V_{u,rv}$ | 40.8 | mL | [1] |
| $E_{max,rv,0}$ | 0.7570 | mmHg/mL | estimated |
| $k_{R,rv}$ | 1.4e-3 | s/mL | [1] |

1. S.6 Afferent input parameters (baroreceptors)

| **Parameter** | **Value** | **Units** | **Reference** |
| --- | --- | --- | --- |
| $P_{n}$ | 92 | mmHg | [1] |
| $f_{min}$ | 2.52 | Hz | [1] |
| $f_{max}$ | 47.78 | Hz | [1] |
| $k_{a}$ | 11.758 | mmHg | [2] |
| $\tau_{z}$ | 6.37 | sec | [1] |
| $\tau_{p}$ | 2.076 | Sec | [1] |

1. S.7 Afferent input parameters (cardiopulmonary receptors)

| **Parameter** | **Value** | **Units** | **Reference** |
| --- | --- | --- | --- |
| $P_{tn}$ | 10.80 | mmHg | [2] |
| $f_{max.l}$ | 20 | Hz | [1] |
| $k_{l}$ | 11.758 | mmHg | [2] |
| $\tau_{cp}$ | 2 | sec | [2] |

1. S.8 Afferent firing frequency gains (to heart)

| **Parameter** | **Value** | **Units** | **Reference** |
| --- | --- | --- | --- |
| $G_{ab,h}$ | 1 | -- | [1] |
| $G_{al,h}$ | 1.541 | -- | Estimated* |
| $G_{ac,h}$ | 2 | -- | [2] |

* Note: $G_{al,h}$ is multiplied by a gain factor of -1 in the Simulink model indicating the “inhibiting” effect lung stretch receptors have on the heart.

1. S.9 Afferent firing frequency gains (to peripheral circulation)

| **Parameter** | **Value** | **Units** | **Reference** |
| --- | --- | --- | --- |
| $G_{ab,p}$ | 1 | -- | [1] |
| $G_{al,p}$ | 0.33 | -- | [4] |
| $G_{ac,p}$ | 2.5 | -- | [2] |

1. S.10 Afferent firing frequency gains (to unstressed volumes)

| **Parameter** | **Value** | **Units** | **Reference** |
| --- | --- | --- | --- |
| $G_{ab,v}$ | 1 | -- | [1] |
| $G_{al,v}$ | 0 | -- | [2] |
| $G_{ac,v}$ | 0 | -- | [2] |

1. S.11 Efferent sympathetic outflow parameters

| **Parameter** | **Value** | **Units** | **Reference** |
| --- | --- | --- | --- |
| $f_{es,0}$ | 16.11 | Hz | [1] |
| $f_{es,\infty}$ | 2.1 | Hz | [1] |
| $f_{es,min}$ | 2.66 | Hz | [1] |
| $k_{es}$ | 0.0675 | Sec | [1] |

1. S.12 Effector function regulation (gains)

| **Parameter** | **Value** | **Units** | **Reference** |
| --- | --- | --- | --- |
| $G_{R,sp}$ | 0.695 | mmHg*mL^-1^*ν^-1^ | [1] |
| $G_{R,ep}$ | 0.653 | mmHg*mL^-1^*ν^-1^ | [1] |
| $G_{R,mp}$ | 2.81 | mmHg*mL^-1^*ν^-1^ | [2] |
| $G_{Vu,sv}$ | -265.4 | mL/ν | [1] |
| $G_{Vu,ev}$ | -107.5 | mL/ν | [2] |
| $G_{Vu,mv}$ | -25 | mL/ν | [2] |
| $G_{T,s}$ | -0.13 | ν | [1] |
| $G_{T,v}$ | 0.09 | ν | [1] |
| $G_{s,Emax,lv}$ | 0.103 | mmHg*mL^-1^*ν^-1^ | estimated |
| $G_{v,Emax,lv}$ | 0.205 | mmHg*mL^-1^*ν^-1^ | estimated |

Where ν = spikes/s (i.e. Hz)

1. S.13 Effector function (time constants)

| **Parameter** | **Value** | **Units** | **Reference** |
| --- | --- | --- | --- |
| $D_{R,sp}$ | 2 | sec | [1] |
| $D_{R,ep}$ | 2 | sec | [1] |
| $D_{R,mp}$ | 2 | sec | [2] |
| $D_{Vu,sv}$ | 5 | sec | [1] |
| $D_{Vu,sv}$ | 5 | sec | [2] |
| $D_{Vu,mv}$ | 5 | sec | [2] |
| $D_{T,s}$ | 2 | sec | [1] |
| $D_{T,v}$ | 0.2 | sec | [1] |
| $D_{E,s}$ | 2 | sec | estimated |
| $D_{E,v}$ | 0.2 | sec | estimated |

1. S.14 Effector function (constants)

| **Parameter** | **Value** | **Units** | **Reference** |
| --- | --- | --- | --- |
| $R_{sp,0}$ | 2.49 | mmHg*s*mL^-1^ | [1] |
| $R_{ep,0}$ | 0.78 | mmHg*s*mL^-1^ | [1] |
| $R_{mp,0}$ | 4.13 | mmHg*s*mL^-1^ | [2] |
| $V_{u,sv,0}$ | 1435.4 | mL | [1] |
| $V_{u,ev,0}$ | 1247 | mL | [2] |
| $V_{u,mv,0}$ | 290 | mL | [3] |
| $T_{0}$ | 0.58 | sec | [1] |
| $D_{T,v}$ | 0.2 | sec | [1] |
| $E_{max,lv.0}$ | 1.283 | mmHg/mL | estimated |
| $E_{max,rv.0}$ | 0.757 | mmHg/mL | estimated |

1. S.15 Basal-Respiration

| **Parameter** | **Value** | **Units** | **Reference** |
| --- | --- | --- | --- |
| $T_{insp}$ | 1.6 | sec | [3] |
| $T_{resp}$ | 4.0 | sec | [3] |
| $T_{exp}$ | 1.4 | sec | [3] |
| $P_{thor,min}$ | -9 | mmHg | [2] |
| $P_{thor, max}$ | -4 | mmHg | [2] |
| $P_{0}$ | 3.9 | mmHg | [2] |

1. S.15B Exercise-associated Respiration

| **Parameter** | **Value** | **Units** | **Reference** |
| --- | --- | --- | --- |
| $T_{insp}$ | 1.4 | sec | [3] |
| $T_{resp}$ | 4 | sec | [3] |
| $T_{exp}$ | 1.6 | sec | [3] |
| $f_{es,cc}$ | 6.5 | Hz | [2] |
| $A$  $(peak value of intramsuclar pressure )$ | 50 | mmHg | [2] |

**Parasympathetic (vagal) outflow**

1. S.16 Neuronal subtype parameters (baroreceptor-input subtype)

| **Parameter** | **Value** | **Units** | **Reference** |
| --- | --- | --- | --- |
| $f_{min,BR}$ | 0.30 | Hz | estimated |
| $f_{max,BR}$ | 21.50 | Hz | estimated |
| $f_{midpt,BR}$ | 1.76 | Hz | estimated |
| $k_{BR}$ | 2.14 | Hz | estimated |

1. S.17 Neuronal subtype parameters (cardiopulmonary receptor-input subtype)

| **Parameter** | **Value** | **Units** | **Reference** |
| --- | --- | --- | --- |
| $f_{min,CPR}$ | 0.30 | Hz | estimated |
| $f_{max,CPR}$ | 21.50 | Hz | estimated |
| $f_{midpt,CPR}$ | 1.76 | Hz | estimated |
| $k_{CPR}$ | 2.14 | Hz | estimated |

1. S.18 Neuronal subtype parameters (lung-stretch receptor input subtype)

| **Parameter** | **Value** | **Units** | **Reference** |
| --- | --- | --- | --- |
| $f_{min,LSR}$ | 2.75 | Hz | estimated |
| $f_{max,LSR}$ | 31.57 | Hz | estimated |
| $f_{midpt,LSR}$ | 0.96 | Hz | estimated |
| $k_{LSR}$ | 7.52 | Hz | estimated |

Table S.19 Nucleus ambiguus neuronal population parameters (heart rate)

| **Parameter** | **Value** | **Units** | **Reference** |
| --- | --- | --- | --- |
| $f_{min,NA}$ | 4.88 | Hz | estimated |
| $f_{max,NA}$ | 15.78 | Hz | estimated |
| $f_{midpt,NA}$ | 0.74 | Hz | estimated |
| $k_{NA}$ | 2.55 | Hz | estimated |

1. S.20 Nucleus ambiguus neuronal population parameters (contractility)

| **Parameter** | **Value** | **Units** | **Reference** |
| --- | --- | --- | --- |
| $f_{min,NActr}$ | 0.61 | Hz | estimated |
| $f_{max,NActr}$ | 11.00 | Hz | estimated |
| $f_{midpt,NActr}$ | 0.62 | Hz | estimated |
| $k_{NActr}$ | 1.20 | Hz | estimated |

1. S.21 Dorsal motor nucleus neuronal population (contractility)

| **Parameter** | **Value** | **Units** | **Reference** |
| --- | --- | --- | --- |
| $f_{min,DMV}$ | 2.59 | Hz | estimated |
| $f_{max,DMV}$ | 6.66 | Hz | estimated |
| $f_{midpt,DMV}$ | 0.53 | Hz | estimated |
| $k_{DMV}$ | 1.24 | Hz | estimated |

1. S.22 NTS subtype gains to NA and DMV

| **Parameter** | **Value** | **Units** | **Reference** |
| --- | --- | --- | --- |
| $K_{BR, NA}$ | 1.0 | -- | estimated |
| $K_{CPR, NA}$ | 1.0 | -- | estimated |
| $K_{LSR, NA}$ | 1.0 | -- | estimated |
| $K_{BR,DMV}$ | 0.0 | -- | estimated |
| $K_{CPR DMV}$ | 1.0 | -- | estimated |
| $K_{LSR,DMV}$ | 1.0 | -- | estimated |

**References**

[1] M. Ursino, Interaction between carotid baroregulation and the pulsating heart: a mathematical model., Am. J. Physiol. 275 (1998) H1733–H1747.

[2] E. Magosso, S. Cavalcanti, M. Ursino, Theoretical analysis of rest and exercise hemodynamics in patients with total cavopulmonary connection., Am. J. Physiol. Heart Circ. Physiol. 282 (2002) H1018-34. doi:10.1152/ajpheart.00231.2001.

[3] E. Magosso, M. Ursino, Cardiovascular response to dynamic aerobic exercise: A mathematical model, Med. Biol. Eng. Comput. 40 (2002) 660–674.

[4] M. Ursino, E. Magosso, Acute cardiovascular response to isocapnic hypoxia. I. A mathematical model., Am. J. Physiol. Heart Circ. Physiol. 279 (2000) H149–H165.
